# Trends and Projections of the Prevalence of Diabetes Mellitus in Pregnancy and Fetal–Neonatal Metabolic Disorders, 2010–2035: A Nationwide Population-Based Study from Hungary

**DOI:** 10.3390/jcm14165740

**Published:** 2025-08-14

**Authors:** Tímea Csákvári, Diána Elmer, Krisztina Palkovics, Luca Fanni Sántics-Kajos, Bettina Kovács, Kálmán Kovács, József Bódis, Imre Boncz

**Affiliations:** 1 Department of Health Economics and Health Care Management, Institute of Health Insurance, Faculty of Health Sciences, University of Pécs, 33 Landorhegyi Street, H-8900 Zalaegerszeg, Hungary; 2 National Laboratory on Human Reproduction, University of Pécs, 4 Vasvári Pál Street, H-7622 Pécs, Hungary; 3 Institute of Health Insurance, Faculty of Health Sciences, University of Pécs, 3 Vörösmarty Street, H-7621 Pécs, Hungary; 4 Doctoral School of Health Sciences, Faculty of Health Sciences, University of Pécs, 4 Vörösmarty Street, H-7621 Pécs, Hungary; 5 Department of Obstetrics and Gynaecology, Clinical Centre, University of Pécs, 17 Édesanyák Street, H-7624 Pécs, Hungary

**Keywords:** diabetes, pregnancy, gestational diabetes, newborns, prevalence

## Abstract

**Objectives**: Diabetes in pregnancy represents a significant public health concern with established impacts on both maternal and fetal health outcomes. Our aim was to evaluate the epidemiology of diabetes mellitus in pregnancy (DMP) and specific fetal and neonatal transient metabolic disorders (FNTMDs) in Hungary between 2010 and 2024, as well as to project future trends through to 2035. **Methods**: We carried out a quantitative, retrospective study using nationwide real-world data from the Hungarian ‘Pulvita’ Health Data Warehouse. ICD-10 codes O24.0–O24.9 (DMP) and P70.0–P70.9 (FNTMDs) were included. Annual patient numbers, the number of hospital days, and the number of DMP patients per 1000 women aged 15–49, as well as the number of FNTMD patients per 1000 live births, were analyzed with joinpoint regression analysis and different forecasting models to project future prevalence up to 2035. **Results**: Despite a 14.2% decrease in live births, DMP cases increased significantly (54.9% inpatient, 26.6% outpatient), with GDM incidence per thousand reproductive-age women rising by 85.7%. FNTMD cases showed similar trends, with GDM-related infant syndromes more than doubling (154% increase). Projections indicate that DMP prevalence could reach 4.60 per 1000 reproductive-age women by 2035, while FNTMD cases show varying trends between inpatient (increasing) and outpatient (stabilizing) care. **Conclusions**: These findings demonstrate a continuing upward trend in diabetes-related pregnancy complications, despite shorter hospital stays, suggesting an urgent need for enhanced preventive programs and specialized care service planning.

## 1. Introduction

According to the International Diabetes Atlas published in 2025, there are an estimated 588.7 million people living with diabetes worldwide among adults aged 20 to 79. This already alarming figure is further exacerbated by projections indicating a 44.81% increase by 2050, based on current prevalence rates and global trends [1]. While the projected growth rate in Europe is expected to be lower (+10.36%), it is important to highlight that actual observed prevalence has consistently exceeded the estimates presented in previous editions of the International Diabetes Federation (IDF) Diabetes Atlas [2].

Additionally, diabetes mellitus in pregnancy could be a significant public health concern. This term encompasses both pre-existing diabetes and diabetes developing during pregnancy (gestational diabetes mellitus, GDM). Among these conditions, GDM accounts for the largest proportion of cases diagnosed. Global estimates suggest that GDM affects approximately 14% of all pregnancies [3]. GDM prevalence varies across different regions, with higher rates observed in Southeast Asia and lower rates in Europe [1]. The impact of GDM extends beyond the gestational period, as affected women are at higher risk of developing type 2 diabetes mellitus (T2DM) later in life, while their children face an increased likelihood of obesity and metabolic disorders [4,5,6].

In Hungary, the population-based prevalence of T2DM is 7.3% [7], according to pharmaceutical utilization. However, the actual prevalence is likely much higher, as this does not account for individuals with non-pharmacological management (e.g., lifestyle changes) and undiagnosed cases. Among women of reproductive age, 0.3% are diagnosed with diabetes, which in many cases may not have developed during pregnancy but rather pre-existed but was undiagnosed [8].

Several risk factors contribute to the development of gestational diabetes mellitus (GDM), which accounts for the largest proportion of diabetes mellitus in pregnancy (DMP). These include advanced maternal age (over 35 years), obesity (pre-pregnancy BMI ≥ 30), a family history of diabetes, particularly among first-degree relatives, GDM in a previous pregnancy, a history of delivering a macrosomic infant (birth weight > 4.5 kg), certain ethnic backgrounds, the presence of polycystic ovary syndrome (PCOS) associated with insulin resistance, a sedentary lifestyle, poor dietary quality, excessive or rapid gestational weight gain, and maternal smoking [9,10,11,12]. Early identification and management of these risk factors are critical for the prevention of GDM and for reducing maternal and fetal complications, such as preeclampsia, intrauterine fetal demise, preterm birth, and high birth weight [13].

Diabetes mellitus in pregnancy, encompassing both GDM and pre-existing diabetes, poses significant risks to both the mother and the developing fetus [14]. Women with pre-existing diabetes are more likely to deliver infants at increased risk of neonatal hypoglycemia, fetal macrosomia (large for gestational age), congenital anomalies, major morbidity often requiring neonatal intensive care, and perinatal mortality [15]. Similarly, GDM significantly affects offspring health by increasing their risk of various metabolic disorders. Children born to mothers with GDM have a higher likelihood of developing obesity, impaired glucose tolerance, and metabolic syndrome from early childhood [16,17,18]. Overall, the presence of DMP may increase the offspring’s risk of developing diabetes in adulthood by as much as eightfold [19].

As global obesity rates continue to rise and maternal age [20] increases, the prevalence of DMP, including GDM and its complications, is expected to grow, underscoring the urgent need for improved prevention strategies, early detection methods, and accessible treatment options worldwide. These strategies must encompass not only improved screening and treatment protocols but also public health initiatives aimed at promoting healthy lifestyles and reducing risk factors.

### DMP Care in Hungary

The Hungarian healthcare system operates on a social health insurance model, providing universal coverage to citizens. For DMP care, Hungary follows a standardized screening and management protocol. According to the 2023 national clinical guideline on diabetes management, all girls with diabetes over the age of 14 should be informed about the importance of preconception care at the time of diagnosis. This interdisciplinary care—provided at a specialized center—should begin no later than six months prior to conception. As part of the care process, risk assessment, nutritional and lifestyle counselling, and, if necessary, diabetes self-management education are provided, alongside the recommended physical examinations, laboratory tests, and evaluation of metabolic status [8]. For diagnosing GDM, screening is recommended between the 24th and 28th weeks of pregnancy, or between the 16th and 18th weeks for women at high risk, using a 75 g oral glucose tolerance test (OGTT). If GDM is diagnosed, interdisciplinary specialist care and follow-up are required. A repeat OGTT is recommended six weeks after delivery. The guideline also emphasizes the importance of preconception education, beginning as early as the planning phase of pregnancy [8].

Overall, while Hungary has established protocols for DMP care, ongoing efforts are needed to optimize screening rates, patient education, and long-term follow-up across the country. Regular assessment of the patient population is also essential to help the healthcare system to prepare for and adapt to DMP management and its associated complications.

Our aim was to evaluate the epidemiology of diabetes mellitus in pregnancy (DMP) and specific fetal and neonatal transient metabolic disorders (FNTMDs) in Hungary between 2010 and 2024, as well as to project future trends through to 2035. These projections can support healthcare decisionmakers in anticipating future service needs, planning resource allocation, and designing preventive interventions tailored to the expected burden of both DMP and FNTMDs.

## 2. Materials and Methods

### 2.1. Methods

We carried out a quantitative, retrospective study using nationwide real-world data.

Although numerous clinical studies and surveys provide data on the prevalence of DMP (specifically GDM), these are typically limited to specific samples, and therefore, their results cannot be generalized to the entire population. In the case of Hungary, however, the individual-level, nationwide database maintained by the National Health Insurance Fund Management (NHIFA) offers a unique opportunity to conduct comprehensive analyses covering the full population. This enables a more reliable and representative understanding of the prevalence of DMP and its trends over time.

### 2.2. Data

The data were obtained from the ‘Pulvita’ Health Data Warehouse: a nationwide, comprehensive data asset that includes, among other things, information on public outpatient and inpatient care utilization and reimbursement. These data are provided, among others, by the National Health Insurance Fund Administration (NHIFA), which is Hungary’s sole national health insurer, and managed by the National Directorate General for Hospitals (NDGH).

Regarding the traffic data of the database, we analyzed both outpatient and inpatient care between 2010 and 2024 (outpatient care data are only available from 2012 onward). The following ICD-10 codes were included in the analysis: O24.0 (pre-existing insulin-dependent diabetes mellitus); O24.1 (pre-existing non-insulin-dependent diabetes mellitus); O24.2 (pre-existing malnutrition-related diabetes mellitus); O24.3 (pre-existing diabetes mellitus of other specified types); O24.4 (gestational diabetes mellitus); and O24.9 (unspecified diabetes mellitus in pregnancy).

To assess the prevalence of FNTMDs, we analyzed the following ICD-10 codes: P70.0 (syndrome of infant of diabetic mother due to gestational diabetes); P70.1 (syndrome of infant of diabetic mother); P70.2 (neonatal diabetes mellitus); P70.3 (iatrogenic neonatal hypoglycemia); P70.4 (other neonatal hypoglycemia); P70.8 (other transient disorders of carbohydrate metabolism of fetus and newborn); and P70.9 (transient disorder of carbohydrate metabolism of fetus and newborn, unspecified).

Patient numbers were defined as the number of unique social security identification numbers (in Hungarian: TAJ) recorded during the study period. According to the database glossary, ‘patient number’ refers to ‘the count of distinct TAJ identifiers appearing in inpatient or outpatient care within the specified timeframe’. This definition ensures that our prevalence estimates reflect the number of individual patients.

For the conditions listed above, we examined the age groups of patients (in DMP cases: 5–18 years; 19–30 years; 31–40 years; 41–50 years), the annual number of patients, and, for inpatient care, the number of hospital days. Based on the extracted data, we also calculated a new variable: the average length of hospital stays.

We also extracted data from the Hungarian Central Statistical Office for the period between 2010 and 2024, including the total number of live births and the number of women aged 15–49, as well as the average age of women at the birth of their first child. Based on these indicators, we calculated the prevalence and the number of patients reported with ICD-10 codes O24 per 1000 women aged 15–49, as well as the number of newborns reported with ICD-10 codes P70 per 1000 live births.

### 2.3. Statistical Analysis

To analyze the variables that most significantly affected patient numbers, we primarily applied joinpoint regression analysis for the period 2010–2024. As a result, we calculated the annual percentage change (APC), which shows the relative change in a variable, within the shorter trends determined by the joinpoint regression model. The second is the annual average percentage change (AAPC), characterizing the whole time series (2010–2024). The most important benefit of these indicators is that they allow for the comparison of variables measured in different dimensions.

The difference in the slope of each trend was tested using a t test, with significant differences marked in all cases (*p* < 0.05) and confidence intervals at a 95% probability level. The analysis was performed using JoinPoint 4.9.0.0 software [21].

Second, we conducted a time series analysis to examine the temporal trends in the prevalence of gestational diabetes and neonatal metabolic disorders based on annual data from 2010 to 2035.

As a first step, we determined and compiled the raw case numbers (ICD-10 codes O24 and P70), along with the number of cases per 1000 live births and per 1000 women aged 15–49 years, separately for inpatient and outpatient care. These data were organized into a database and imported into SPSS 27.0 software.

In the second step, we calculated the parameters of the autocorrelation (ACF) and partial autocorrelation (PACF) functions for each time series, which were considered in model selection and adjustment (Figure S1). We evaluated the predictive accuracy of candidate models using standard goodness-of-fit indicators, including the mean absolute percentage error (MAPE), root mean square error (RMSE), and coefficient of determination (R^2^). For each time series, we tested and compared multiple models (e.g., ARIMA, Brown’s exponential smoothing, and simple exponential smoothing) and selected the one that yielded the most favorable performance based on these indicators. All forecasts were performed through to the year 2035.

In the forecasting models, we included the years 2020–2021 as event variables to account for the exceptional impact of the COVID-19 pandemic on case numbers.

### 2.4. Ethical Considerations

All data were provided and processed in aggregated form, and individual identification was not possible; therefore, ethical approval was not required for this study.

## 3. Results

In Hungary, a total of 90,335 live births were registered in 2010. During the study period, the highest numbers were recorded in 2016 (93,063) and in 2021 (93,039); however, a decline was shown afterwards.

Between 2010 and 2024, the epidemiological burden of DMP and FNTMDs increased significantly, despite a decline in the number of births and women of reproductive age. While the number of live births decreased by 14.2%, the number of DMP patients increased by 54.9% in inpatient care and by 26.6% in outpatient care. Notably, the incidence of gestational diabetes cases per thousand women of reproductive age increased even more so (85.7%), although the mean length of hospital stays decreased (–29.32%). The number of FNTMD cases also increased: the number of affected newborns per thousand live births rose by nearly 38%, and cases diagnosed as ‘syndrome due to GDM mother’ more than doubled (154%). Overall, the prevalence of the diseases analyzed increased, while the reduction in the length of hospital stays indicates some positive changes in care delivery (Table 1).

Table 2 shows the changes in the trends observed in the population’s basic demographic data. Between 2010 and 2024, the number of women of reproductive age (15–49 years) decreased by 11.45%, with an average annual decrease of 0.9%. Although the downward trend has persisted since 2021, the rate of decline appears to have slightly slowed.

A clear turning point was also observed in 2021 regarding the number of live births per 1000 women of reproductive age. Prior to 2021, a significant upward trend was evident, with an average annual increase of 1.2%. However, after 2021, this indicator began to decline considerably, with an average annual decrease of 4.3%.

An upward trend can also be seen in the average age of mothers at the birth of their first child. In Hungary, this age was 28.23 years in 2010 and increased to 29.43 years by 2024, with a more pronounced rise beginning in 2014 (Table 2).

**Figure 1:**
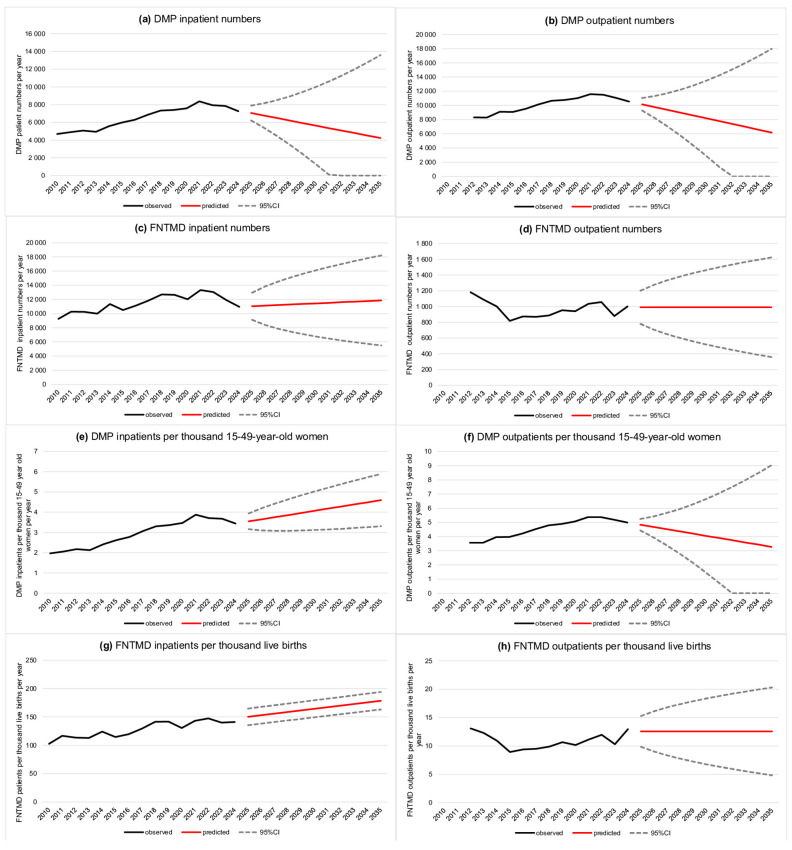
Results of the forecasting models: (**a**) crude DMP patient numbers in inpatient care; (**b**) crude DMP patient numbers in outpatient care; (**c**) crude FNTMD patient numbers in inpatient care; (**d**) crude FNTMD patient numbers in outpatient care; (**e**) DMP patients per thousand 15–49-year-old women (inpatient care); (**f**) DMP patients per thousand 15–49-year-old women (outpatient care); (**g**) FNTMD patients per thousand live births (inpatient care); and (**h**) FNTMD patients per thousand live births (inpatient care). DMP—diabetes mellitus in pregnancy; FNTMD—fetal and neonatal transient metabolic disorders.

During the study period, a total of 98,086 patients were treated for gestational diabetes in inpatient care, with an annual average of 6539 patients; 91.44% of them were diagnosed with GDM. The highest number of patients was recorded in 2021 (n = 8371), while the lowest annual case number was observed in 2010 (n = 4693). Given that a generally decreasing annual case number has been observed since 2021, the model predicts a continued decline in case numbers, although with a wide confidence interval (Figure 1a).

Between 2010 and 2024, a total of 131,574 patients were registered in outpatient care with DMP, an average of 10,121 patients per year. The highest number was recorded in 2021 (n = 11,584), while the lowest was recorded in 2013 (n = 8274). Based on the trends projected by the model, a decline in outpatient numbers is anticipated, potentially decreasing to as few as 6178 patients by 2035 (Figure 1b).

In 2010, a total of 9276 newborns were registered with FNTMDs in inpatient care. On average, 37% of cases were classified as syndromes of infants of mothers with GDM (P70.0) and another 37% as ‘other transitory disorders of carbohydrate metabolism of fetus and newborn’ (P70.8). The assessed time series peaked in 2021, with 13,332 cases, followed by a steady annual decline. In outpatient care, the case numbers are approximately between 800 and 1000 each year, with the highest number in 2012 (n = 1182) and the lowest in 2015 (n = 819). According to our results, the absolute indicators are expected to remain stable over the projected period, with a slight increase anticipated in inpatient care (Figure 1c,d).

Given that the declining population may also influence the above-mentioned indicators, we also examined the number of DMP cases per 1000 women aged 15–49, as well as the number of newborns with FNTMDs per 1000 live births. These indicators already exhibit an increasing trend, and further growth is projected between 2025 and 2035 according to our models. In 2010, there were 1.97 DMP cases per 1000 women of reproductive age, rising to 3.44 by 2024. Our projections indicate that this figure could increase to 4.60 by 2035 (Figure 1e). In contrast, the number of outpatients may decline, although the predicted time series is uncertain in this case (Figure 1f). As for FNTMDs, our model suggests an increase in the inpatient care figures within the target population (Figure 1g), while a plateau is expected in outpatient care between 2025 and 2035 (Figure 1h).

Furthermore, we analyzed the patient numbers for each individual disorder (ICD code) separately (Figure S2). Due to the smaller sample sizes in some groups, clear trends could not be established; however, for conditions of greater public health significance (e.g., GDM or syndrome of infant of mother with GDM), a clear upward trend is observed in the absolute numbers.

## 4. Discussion

Our main objective was to assess the number and population proportion of women affected by diabetes during pregnancy, as well as newborns diagnosed with metabolic disorders, and to project trends expected over the next decade in Hungary. Our data were determined comprehensively, with nationwide coverage, for the period 2010–2035, separately based on utilization indicators of outpatient and inpatient care.

The proportion of women diagnosed with DMP increased from 1.97‰ to 3.44‰ over the study period. Our findings are consistent with previous Hungarian studies, in which the prevalence of diabetes among women of reproductive age was estimated at 0.3% [8]. A meta-analysis reported a prevalence of 0.5% for pre-existing diabetes in pregnancy across European countries [22]. Although comparable data on DMP prevalence across Central and Eastern Europe are limited, recent statistics on age-adjusted diabetes prevalence provide valuable regional context. According to the latest report of the IDF, neighboring countries such as Croatia and Serbia have reported diabetes prevalence rates of 10.5%, while Romania (7.5%), Slovakia (7.1%), and Slovenia (7.0%) show moderately high rates. Hungary’s age-adjusted prevalence stands at 8.3%, indicating a considerable burden of disease. In contrast, countries such as Austria (5.4%) and Ukraine (6.0%) report lower rates. Given the influence of shared socioeconomic and healthcare system factors across these countries, it is likely that DMP has followed similar trends in the region. These parallels underscore the importance of a regional public health approach to DMP [1,23]. Given the shared socioeconomic and healthcare characteristics of the region and the strong pathophysiological link between T2DM and gestational diabetes, it is likely that the prevalence of DMP has followed a comparable upward trajectory in the region.

In studies involving universal screening, early neonatal hypoglycemia rates ranged from 3.4% to 12.1%, with one study reporting a rate of 4.1 per 1000 live births [24]. According to our own findings, the prevalence of FNTMDs in Hungary in 2024 was 14.1% in inpatient and 1.30% in outpatient care. Gestational age, birth weight extremes (small and large for gestational age), and maternal diabetes consistently emerge as key risk factors [25,26].

Among our key findings is that, although the raw number of DMP cases shows a declining trend across both levels of care, our models project an increase in population-adjusted indicators—specifically, the number of DMP cases per 1000 women aged 15–49. From a healthcare system perspective, this suggests that a growing proportion of women in the childbearing population are affected by these conditions and may require monitoring or intervention. This trend highlights the importance of maintaining preparedness in maternal and neonatal care services, even in the context of declining birth rates.

The raw number of FNTMD cases per 1000 live births is expected to increase in inpatient care and remain stable in outpatient care over the coming period. Similar trends are anticipated for the corresponding per 1000 live births indicators as well.

A notable discrepancy was observed between the number of inpatient and outpatient FNTMD cases, with inpatient diagnoses consistently exceeding 13,000 cases annually, while outpatient figures remained below 1200. This divergence may be clinically justified, as fetal and neonatal transient metabolic disorders typically present acutely in the immediate postnatal period, often necessitating short-term inpatient management. These conditions are generally resolved before discharge and may not require further follow-up within the same ICD-10 category in outpatient settings.

In our time series analysis, both the number of live births and the number of cases peaked in 2021 in several instances. A possible explanation for this is the COVID-19 pandemic, as studies have reported an increase in the incidence of GDM during the pandemic period [27,28], findings that align with our own observations. Potential contributing factors include a more sedentary lifestyle, poor dietary habits, and limited access to healthcare services. To mitigate the potential confounding effect of COVID-19, we modeled the years 2020 and 2021 as event variables in the time series analysis.

Several interrelated factors may underlie our findings. On the one hand, known risk factors—such as increasing maternal age and rising prevalence of overweight and obesity, as well as the spread of physical inactivity and unhealthy dietary habits—are increasingly common among women of reproductive age. An increase in the frequency of family history and lifestyle factors predisposing to insulin resistance, along with socioeconomic and healthcare-system-level influences, may also contribute. With the reduction in regional disparities, improvements in certain components of the healthcare system, and enhanced quality of centralized data collection (e.g., NHIFA), more cases may be registered, which statistically manifests as an upward trend.

Global diabetes (and DMP) prevalence is rising and is expected to continue increasing, particularly in developing countries due to urbanization, sedentary lifestyles, and changing dietary habits [1]. Geographic disparities are notable, with Southeast Asia, the Middle East, and Africa likely experiencing more pronounced growth as social and lifestyle changes occur rapidly. Additionally, T2DM is anticipated to affect younger age groups as well [29]. Socioeconomic factors also play a critical role, as lower-income communities face higher rates of metabolic disorders due to limited access to healthcare and health-promoting resources [30]. Technological advancements, especially improvements in screening methods and diagnostic criteria, contribute to increased case detection [31,32]. The global obesity epidemic further exacerbates diabetes and DMP risks. All these trends underscore the urgent need to strengthen prevention strategies, ensure accessible and high-quality healthcare, and implement targeted interventions to reduce the worldwide burden of diabetes. In this context, the role of existing national guidelines becomes important. In Hungary, the 2023 clinical guideline outlines comprehensive protocols for early screening, multidisciplinary care, and postpartum follow-up, with particular attention to high-risk groups [8]. While this framework offers a solid foundation, its long-term impact may depend on sustained efforts to enhance health literacy among women of reproductive age and to support consistent application across all levels of care. As the next edition of the national guideline is expected to be released in 2026, incorporating the latest international evidence and addressing emerging challenges could further strengthen preventive strategies and health service responses.

The strength of this study lies in the use of comprehensive, nationwide health administrative data that reflect real-world clinical practice. Consequently, the analysis is not only representative but also interpretable at the population level, which is particularly important for assisting public health decision-making.

However, the study faced several limitations. The period analyzed was relatively short, especially regarding predictive modeling, necessitating cautious interpretation of long-term forecasts. While conservative extrapolation methods were applied and the results were cross-validated using multiple model specifications, projections extending beyond 10 years should be interpreted with particular caution. Given the limited historical timeframe (2010–2024), the underlying assumptions of stationarity and long-term model validity may not hold.

In addition to the direct impact of the COVID-19 pandemic, other external factors may have influenced the observed trends between 2010 and 2024. Additional factors, such as changes in public awareness of gestational diabetes, healthcare access, or broader lifestyle-related trends (e.g., increasing obesity rates, dietary habits, and sedentary behavior), may also have contributed to the observed prevalence rates. Although our dataset did not include variables to directly assess these potential confounders, acknowledging their possible influence is important when interpreting the results, and they should be considered in future studies. This may limit the comprehensive interpretation of the results, particularly in understanding the underlying causes of the observed changes. Finally, it is important to note that the structure of the predictive model itself may contribute to the observed increasing trends; if the model does not incorporate parameters accounting for the effects of future preventive or mitigating interventions, the projected values may overestimate the likely future prevalence.

Also, one must interpret the observed trends with caution, as the data may be affected by potential coding biases. A significant proportion of cases, especially in FNTMD cases, were classified under ‘other’ or ‘unspecified’ categories, which may indicate diagnostic uncertainty, variability in clinical documentation practices, or differences in coding accuracy over time. These factors may hide real epidemiological trends and complicate comparisons across years. Moreover, they may affect the validity of the projections by introducing systematic error or noise into the input data, potentially leading to over- or underestimation of future trends. The observed decrease in unspecified diagnoses in some categories may reflect improvements in diagnostic precision, but it may also result from shifts in coding habits rather than actual changes in disease incidence.

## 5. Conclusions

Although the absolute number of DMP cases shows a decline in both outpatient and inpatient care, population-adjusted analyses project the opposite: the prevalence among women aged 15–49 is likely to increase. Concurrently, the number of FNTMD cases is not expected to decrease significantly; inpatient data indicate an upward trend, while outpatient care is anticipated to remain stable.

This highlights that diabetes during pregnancy and its consequences will continue to pose a significant public health challenge. It is therefore warranted for policymakers to support the implementation of existing preventive programs, with particular emphasis on strengthening targeted interventions aimed at maternal metabolic health. Furthermore, the presented projections should play a crucial role in planning the capacity and accessibility of specialized care services. To effectively mitigate the projected trends, particular attention should be given to strengthening interdisciplinary collaboration among healthcare providers responsible for education and care of women with DMP, as well as ensuring adequate neonatal care resources, timely postnatal screening, and the availability of trained neonatology and metabolic care professionals. According to current national guidelines, preconception and prenatal care of women with diabetes requires coordinated efforts by health visitors, physicians, diabetes specialist nurses, and general practitioners. Their joint work, supported by continuous professional training, is essential to improving both prevention and care outcomes.

## Figures and Tables

**Table 1:** Descriptive statistics.

Variables	2010 ^a^	2024	Change (%)
Number of live births (n)	90,335	77,500	−14.21%
Mean age of women at first childbirth (years)	28.23	29.43	4.25%
Number of 15–49-year-old women (n)	2,385,782	2,112,676	−11.45%
Number of live births per thousand 15–49-year-old women (n)	37.86	36.68	−3.10%
Number of DMP patients (inpatient care) (n) *Gestational* *Pre-existing* *Unspecified*	4693 *4168 (88.81%)* *334 (7.12%)* *191 (4.07%)*	7271 *6853 (94.25%)* *370 (5.09%)* *48 (0.66%)*	54.93% *64.42%* *10.78%* *−74.87%*
Number of DMP patients per thousand 15–49-year-old women (inpatient care) (n) *Gestational* *Pre-existing* *Unspecified*	1.97 *1.75* *0.14* *0.08*	3.44 *3.24* *0.18* *0.02*	74.96% *85.67%* *25.10%* *−71.62%*
Age distribution in inpatient care (n, [%])			
5–18 years *Gestational* *Pre-existing* *Unspecified*	37 *32 (86.49%)* * 5 (13.51%)* -	42 40 (95.24%) 2 (4.76%) -	13.51% *25.00%* *−60.00%* -
19–30 years *Gestational* *Pre-existing* *Unspecified*	1858 *1662 (89.45%)* *131 (7.05%)* *65 (3.50%)*	2729 *2578 (94.47%)* *132 (4.84%)* *19 (0.70%)*	46.88% *55.11%* *0.76%* *−70.77%*
31–40 years *Gestational* *Pre-existing* *Unspecified*	2649 *2340 (88.34%)* *187 (7.06%)* *122 (4.61%)*	4085 *3842 (94.05%)* *216 (5.29%)* *27 (0.66%)*	54.21% *64.19%* *15.51%* *−77.87%*
41–50 years *Gestational* *Pre-existing* *Unspecified*	149 *134 (89.93%)* *11 (7.38%)* *4 (2.68%)*	415 *393 (94.70%)* *20 (4.82%)* *2 (0.48%)*	178.52% *193.28%* *81.82%* *−50.00%*
DMP mean length of hospital stays (days) *Gestational* *Pre-existing* *Unspecified*	8.43 *7.99* *10.44* *12.34*	5.96 *5.86* *10.02* *5.23*	−29.32% *−26.66%* *−4.02%* *−57.62%*
Number of DMP patients (outpatient care) (n) *Gestational* *Pre-existing* *Unspecified*	8319 *7098 (85.32%)* *681 (8.19%)* *540 (6.49%)*	10,530 *9513 (90.34%)* *558 (5.30%)* *459 (4.36%)*	26.58% 34.02% −18.06% −15.00%
Number of DMP patients per thousand 15–49-year-old women (outpatient care) (n) *Gestational* *Pre-existing* *Unspecified*	3.56 *3.04* *0.29* *0.23*	4.98 *4.50* *0.26* *0.22*	39.84% *48.03%* *−10.34%* *−4.35%*
Number of FNTMD patients (inpatient care) (n) *Syndrome due to GDM mother* * Syndrome due to diabetic mother* *Neonatal diabetes* *Neonatal hypoglycemia* *Other, unspecified*	9276 *2250 (24.26%)* *289 (3.12%)* *4 (0.04%)* *3214 (34.65%)* *3519 (37.94%)*	10,951 *4909 (44.83%)* *300 (2.74%)* *4 (0.04%)* *1537 (14.04%)* *4201 (38.36%)*	18.06% 118.18% 3.81% 0.00% −52.18% 19.38%
Number of FNTMD patients per thousand live births (inpatient care) (n) *Syndrome due to GDM mother* * Syndrome due to diabetic mother* *Neonatal diabetes* *Neonatal hypoglycemia* *Other, unspecified*	102.68 *24.91* *3.20* *0.04* *35.58* *38.96*	141.30 *63.34* *3.87* *0.05* *19.83* *54.21*	37.61% *154.28%* *20.94%* *25.00%* *−44.27%* *39.14%*
**FNTMD** **mean length of hospital stays (days)** *Syndrome due to GDM mother* * Syndrome due to diabetic mother* *Neonatal diabetes* *Neonatal hypoglycemia* *Other, unspecified*	6.94 *4.62* *5.18* *11.67* *5.75* *7.29*	5.24 *3.60* *4.05* - *6.31* *6.92*	−24.47% *−22.08%* *−21.81%* - *9.74%* *−5.08%*
**Number of** **FNTMD** **patients (outpatient care) (n)** *Syndrome due to GDM mother* * Syndrome due to diabetic mother* *Neonatal diabetes* *Neonatal hypoglycemia* *Other, unspecified*	1182 *518 (43.82%)* *103 (8.71%)* *4 (0.34%)* *398 (33.67%)* *159 (13.45%)*	1003 *177 (17.65%)* *43 (4.29%)* *3 (0.30%)* *176 (17.55%)* *604 (60.22%)*	−15.14% −12.66% −58.25% −25.00% −55.78% −279.87%
**Number of** **FNTMD** **patients per thousand live births (outpatient care) (n)** *Syndrome due to GDM mother* * Syndrome due to diabetic mother* *Neonatal diabetes* *Neonatal hypoglycemia* *Other, unspecified*	13.09 *5.74* *1.14* *0.04* *4.41* *1.76*	12.94 *2.28* *0.55* *0.04* *2.27* *7.79*	−1.16% *−60.28%* *−51.75%* *0.00%* *−48.53%* *342.61%*

^a^ 2012 for outpatient variables; DMP—diabetes mellitus in pregnancy; GDM—gestational diabetes mellitus; FNTMD—fetal and neonatal transient metabolic disorders.

**Table 2:** Results of the joinpoint regression analysis for the base variables.

Variable	AAPC (95%CI)	Trends			
		Trend 1		Trend 2	
		APC (95%CI)	Period	APC (95%CI)	Period
Number of live births	−0.9 *	0.3	2010–2021	−5.1 *	2021–2024
	(−1.5–−1.3)	(−0.1–0.7)		(−7.7–−2.4)	
Number of live births per 1000 15–49-year-old women	0.0	1.2 ***	2010–2021	−4.3 **	2021–2024
	(−0.6–0.6)	(0.9–1.6)		(−7.0–−1.6)	
Mean age of women at first childbirth	0.3 *	0.2	2010–2014	0.4 ***	2014–2024
	(0.2–0.3)	(−0.2–0.2)		(0.3–0.4)	
Number of 15–49-year-old women	−0.9 *	−0.9 *	2010–2021	−0.7 *	2021–2024
	(−0.9–−0.8)	(−0.9–−0.9)		(−1.0–−0.4)	

AAPC: Annual average percentage change; ***: significant at *p* < 0.001; **: significant at *p* < 0.01; *: significant at *p* < 0.05.

## Data Availability

Restrictions apply to the availability of these data. Data were obtained from the National Directorate General for Hospitals (NDGH) of Hungary and are available from the authors with the permission of the NDGH.

## Supplementary Materials

The following supporting information can be downloaded at https://www.mdpi.com/article/10.3390/jcm14165740/s1: Figure S1: Model diagnostics: ACF and PACF of residuals from all models. Figure S2: Projected trends for diagnostic subgroups of DMP and FNTMD patient numbers.

## Abbreviations

The following abbreviations are used in this manuscript:

AAPC	Annual average percentage change
APC	Annual percentage change
DMP	Diabetes mellitus in pregnancy
FNTMDs	Fetal and neonatal transient metabolic disorders
GDM	Gestational diabetes mellitus
NDGH	National Directorate General for Hospitals
NHIFA	National Health Insurance Fund Administration
