# Supplementary material for: Trends and Projections of the Prevalence of Diabetes Mellitus in Pregnancy and Fetal–Neonatal Metabolic Disorders, 2010–2035: A Nationwide Population-Based Study from Hungary
Source: J Clin Med. 2025 Aug 14;14(16):5740. doi: 10.3390/jcm14165740 (PMC12387990; doi:10.3390/jcm14165740)
Supplement: Supplementary file 1 [file jcm-14-05740-s001.zip › Csákvári_gest_diabetes_Figure_S2_20250623.pdf]

Number of DMP patients by ICD-10 codes (inpatient care)

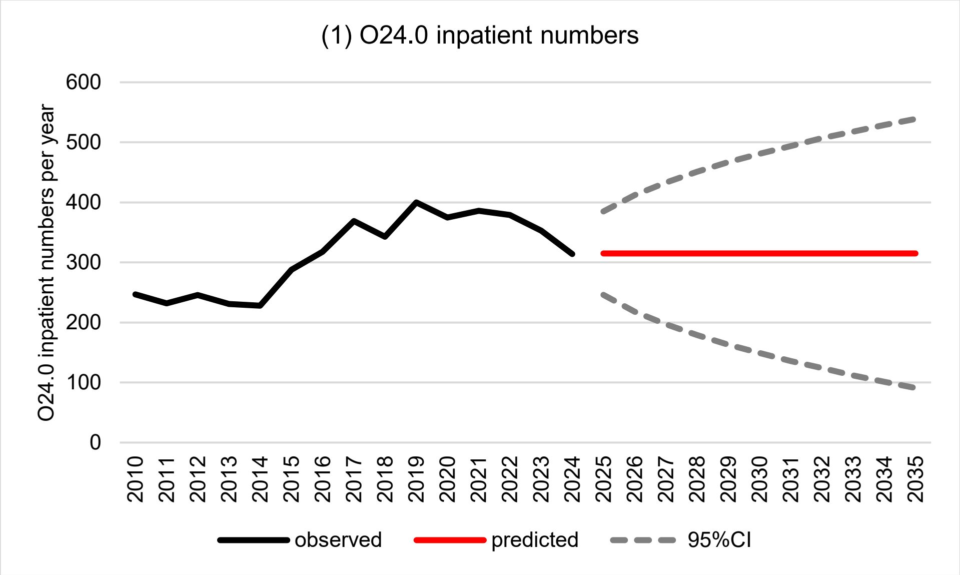

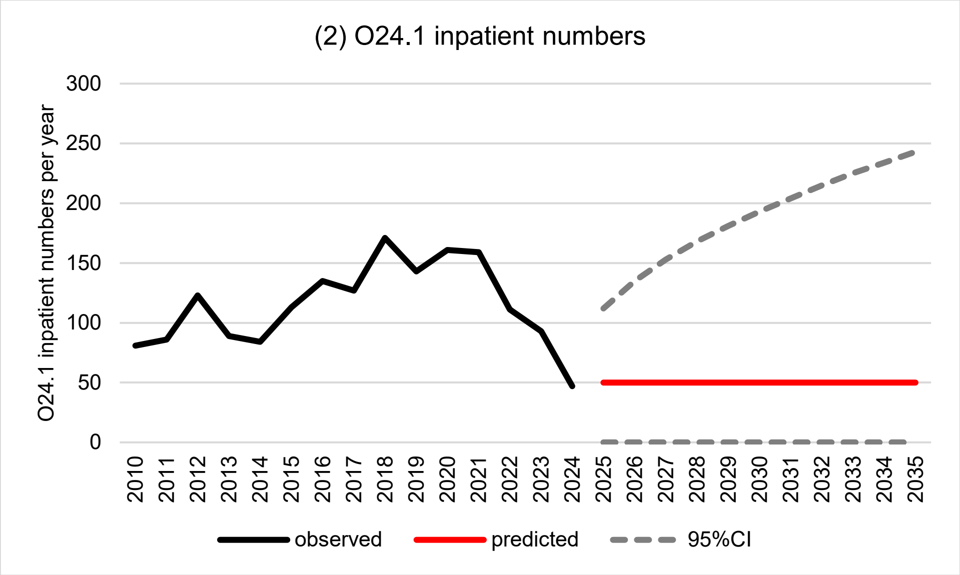

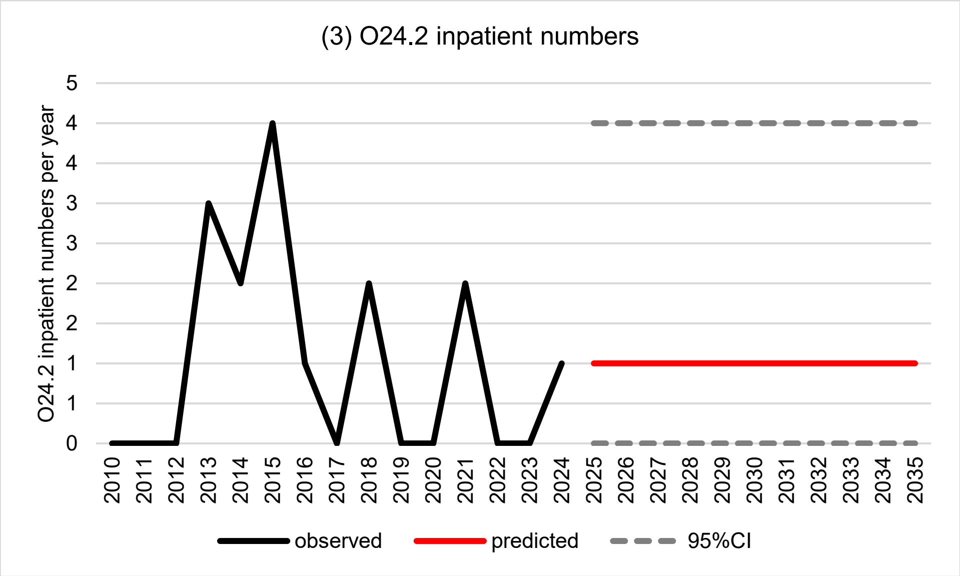

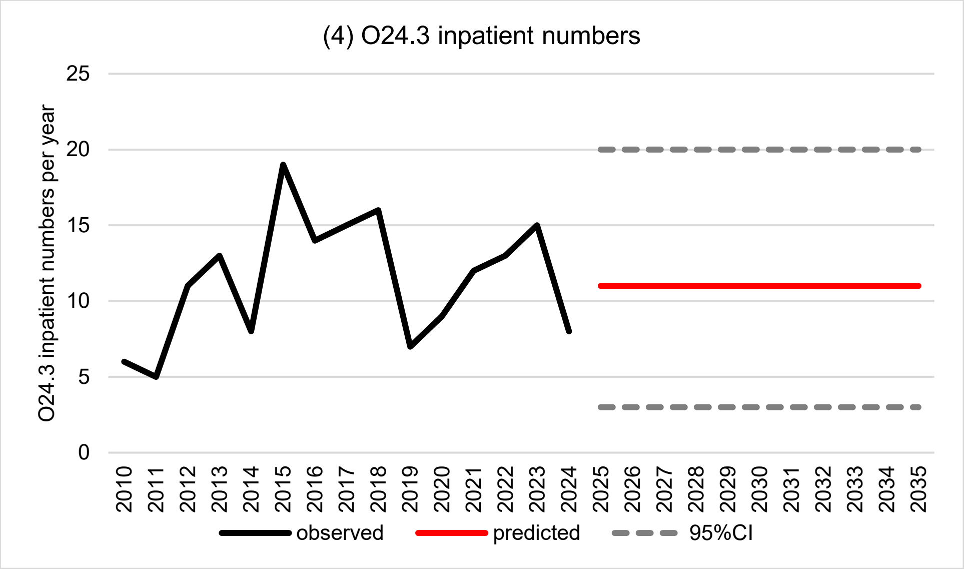
(4) O24.3 inpatient numbers
O24.3 inpatient numbers per year
25
20
15
10
5
0
2010
2011
2012
2013
2014
2015
2016
2017
2018
2019
2020
2021
2022
2023
2024
2025
2026
2027
2028
2029
2030
2031
2032
2033
2034
2035
observed
predicted
95%CI

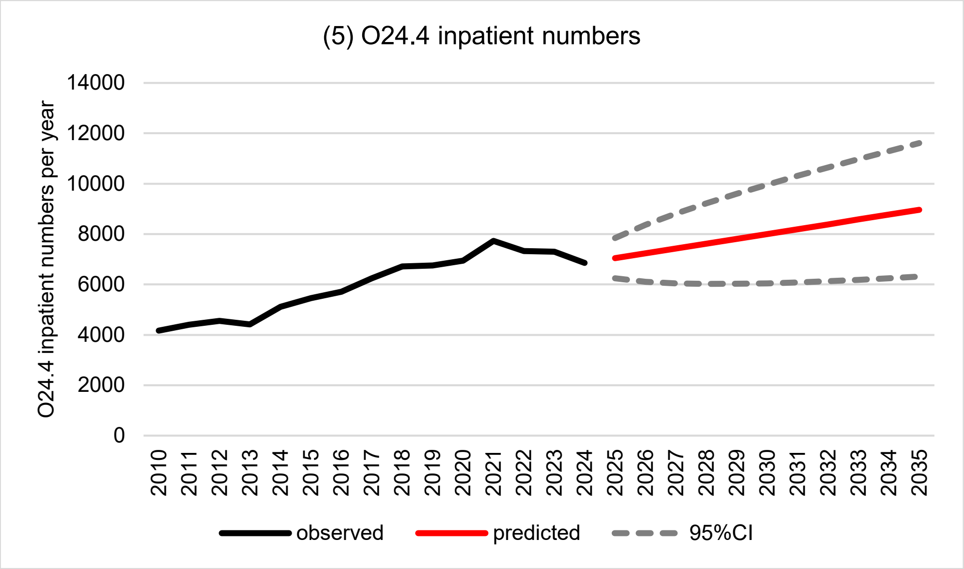
(5) O24.4 inpatient numbers
O24.4 inpatient numbers per year
14000
12000
10000
8000
6000
4000
2000
0
2010
2011
2012
2013
2014
2015
2016
2017
2018
2019
2020
2021
2022
2023
2024
2025
2026
2027
2028
2029
2030
2031
2032
2033
2034
2035
observed
predicted
95%CI

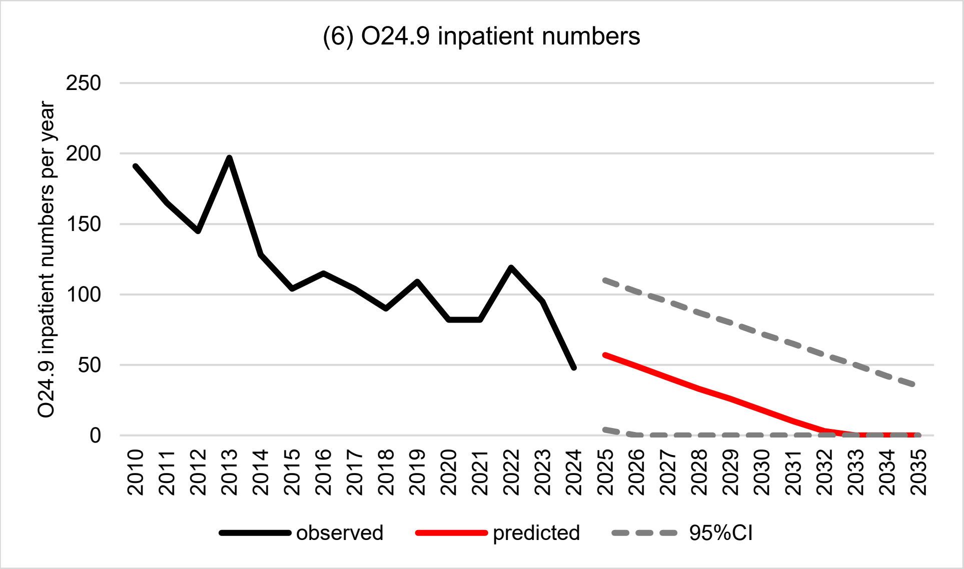
(6) O24.9 inpatient numbers
O24.9 inpatient numbers per year
250
200
150
100
50
0
2010
2011
2012
2013
2014
2015
2016
2017
2018
2019
2020
2021
2022
2023
2024
2025
2026
2027
2028
2029
2030
2031
2032
2033
2034
2035
observed
predicted
95%CI

Number of FNTMD patients by ICD-10 codes (inpatient care)

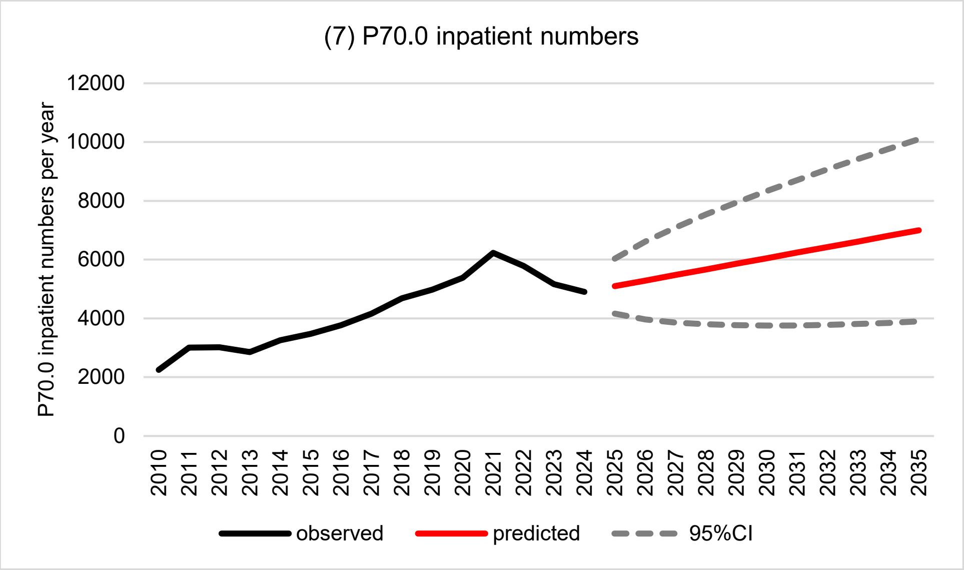

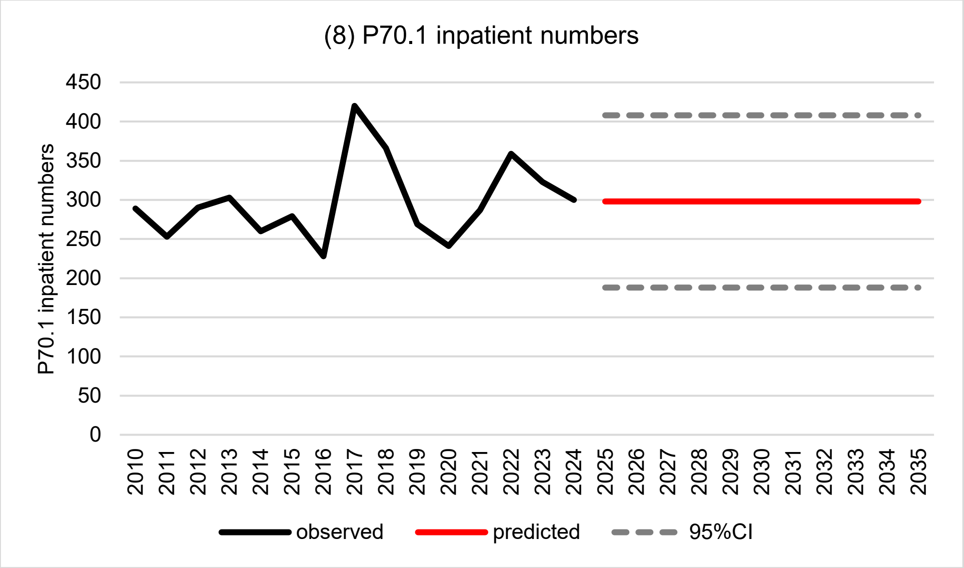

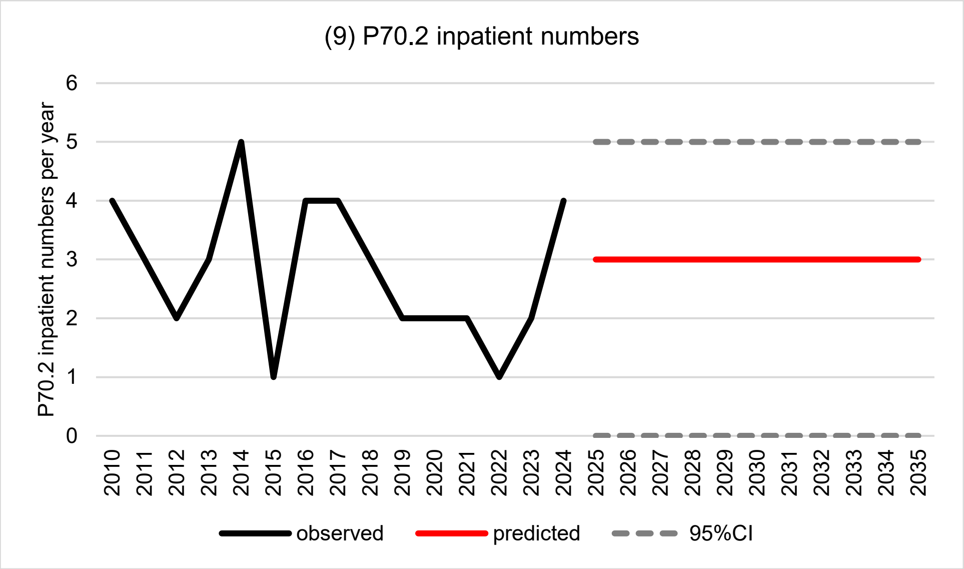

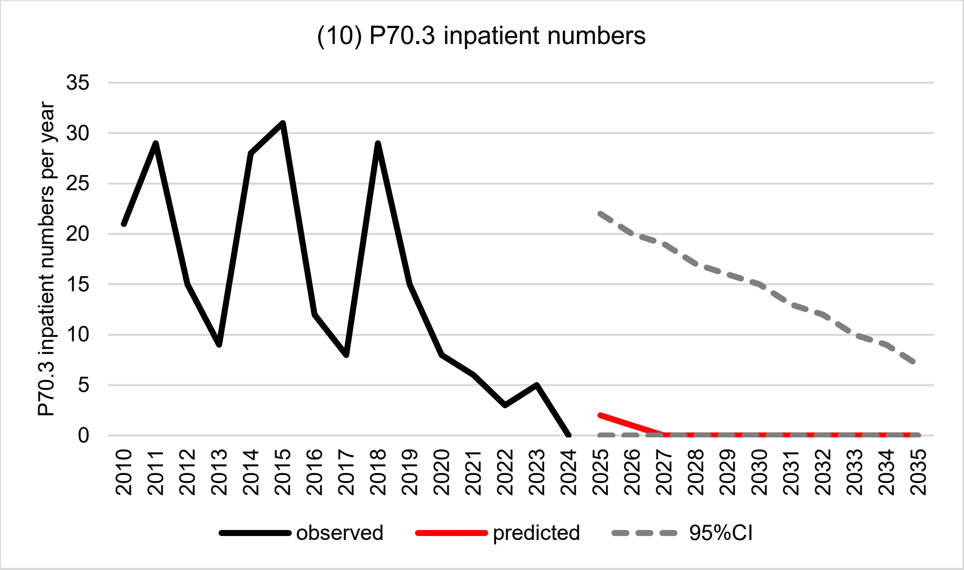
(10) P70.3 inpatient numbers
P70.3 inpatient numbers per year
35
30
25
20
15
10
5
0
2010 2011 2012 2013 2014 2015 2016 2017 2018 2019 2020 2021 2022 2023 2024 2025 2026 2027 2028 2029 2030 2031 2032 2033 2034 2035
observed
predicted
95%CI

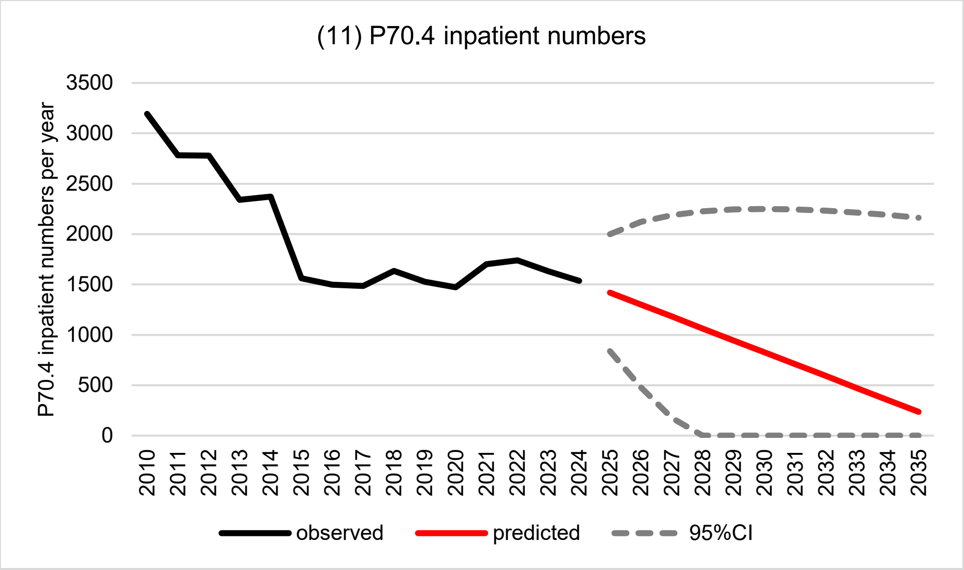
(11) P70.4 inpatient numbers
P70.4 inpatient numbers per year
3500
3000
2500
2000
1500
1000
500
0
2010 2011 2012 2013 2014 2015 2016 2017 2018 2019 2020 2021 2022 2023 2024 2025 2026 2027 2028 2029 2030 2031 2032 2033 2034 2035
observed
predicted
95%CI

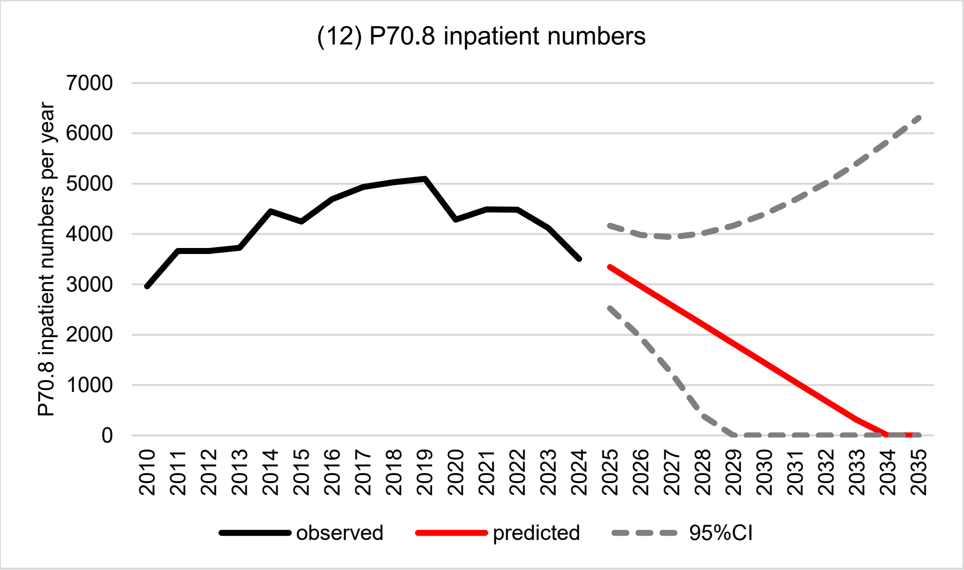
(12) P70.8 inpatient numbers
P70.8 inpatient numbers per year
7000
6000
5000
4000
3000
2000
1000
0
2010 2011 2012 2013 2014 2015 2016 2017 2018 2019 2020 2021 2022 2023 2024 2025 2026 2027 2028 2029 2030 2031 2032 2033 2034 2035
observed
predicted
95%CI

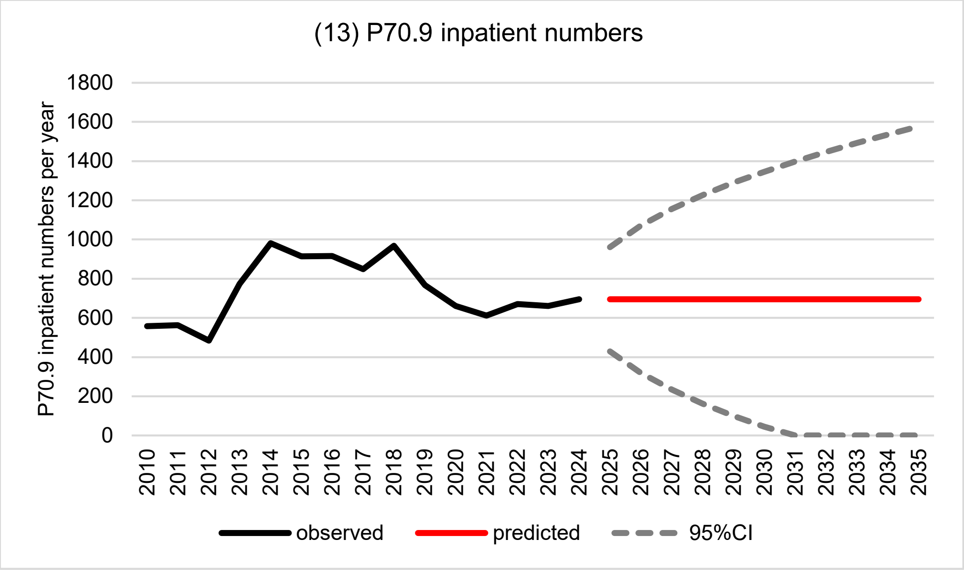
(13) P70.9 inpatient numbers
P70.9 inpatient numbers per year
1800
1600
1400
1200
1000
800
600
400
200
0
2010
2011
2012
2013
2014
2015
2016
2017
2018
2019
2020
2021
2022
2023
2024
2025
2026
2027
2028
2029
2030
2031
2032
2033
2034
2035
observed
predicted
95%CI

Number of DMP patients by ICD-10 codes (outpatient care)

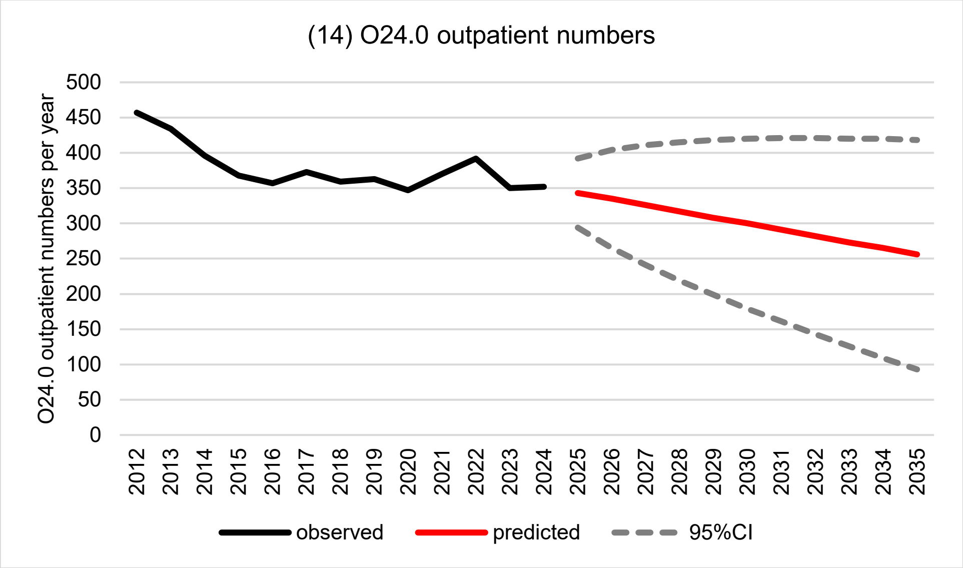

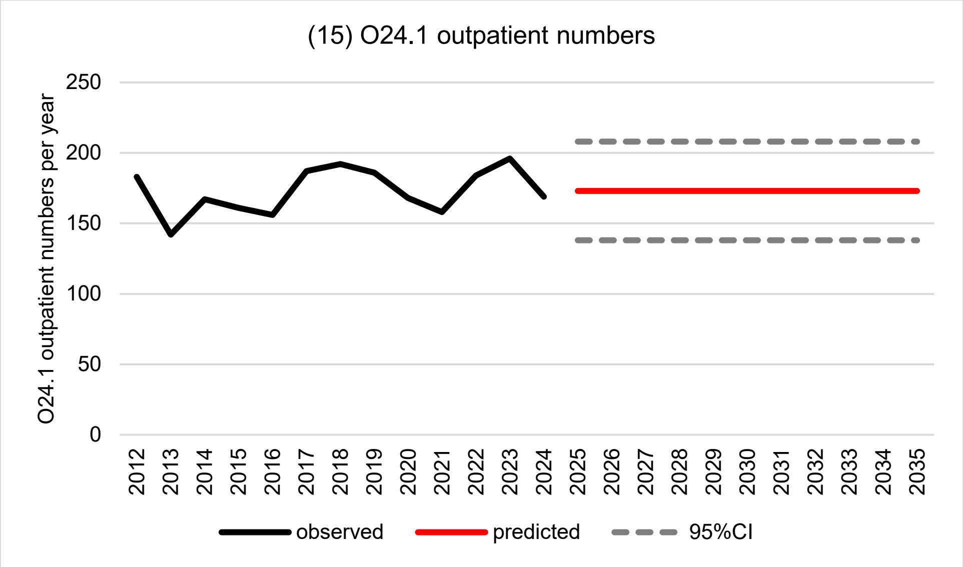

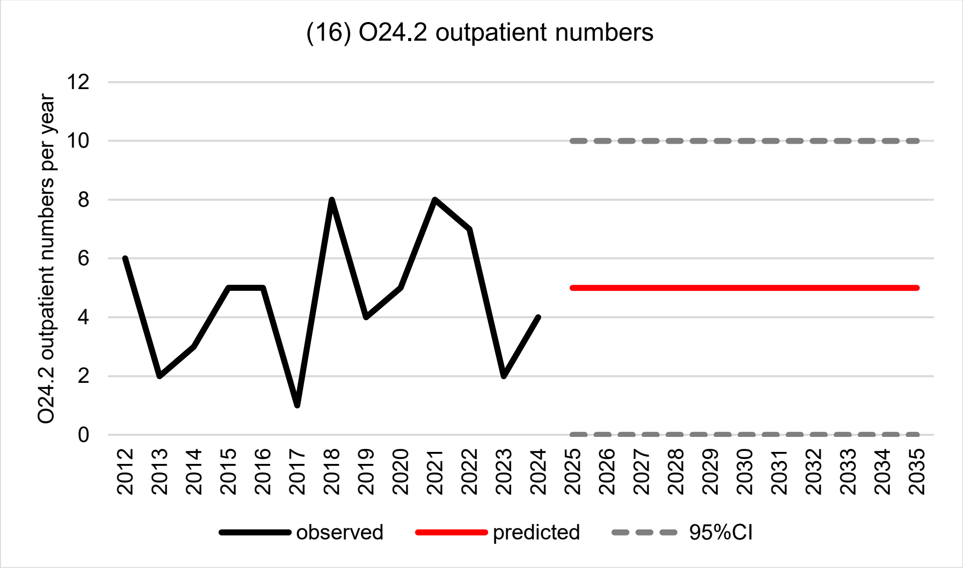

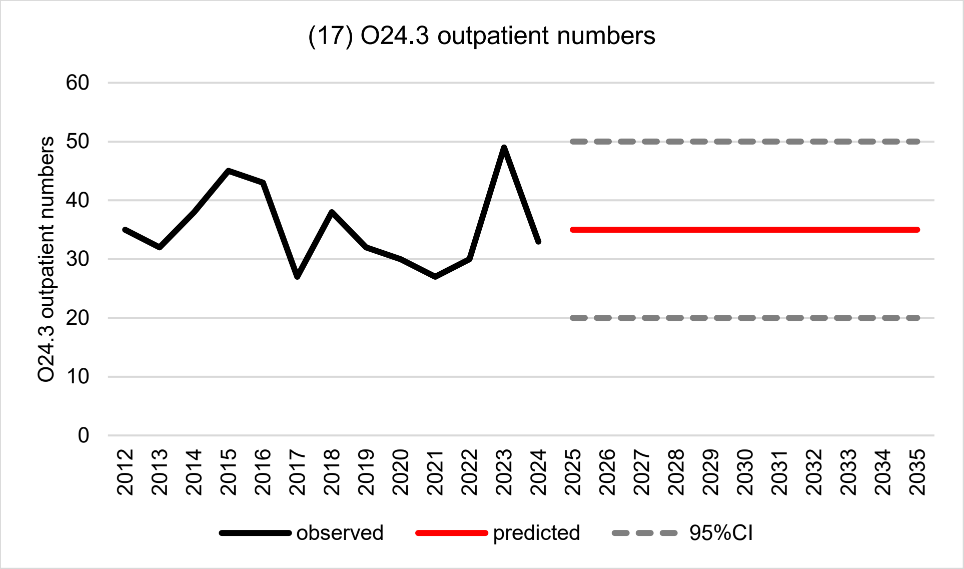

(17) O24.3 outpatient numbers
O24.3 outpatient numbers
60
50
40
30
20
10
0
2012
2013
2014
2015
2016
2017
2018
2019
2020
2021
2022
2023
2024
2025
2026
2027
2028
2029
2030
2031
2032
2033
2034
2035
observed
predicted
95%CI

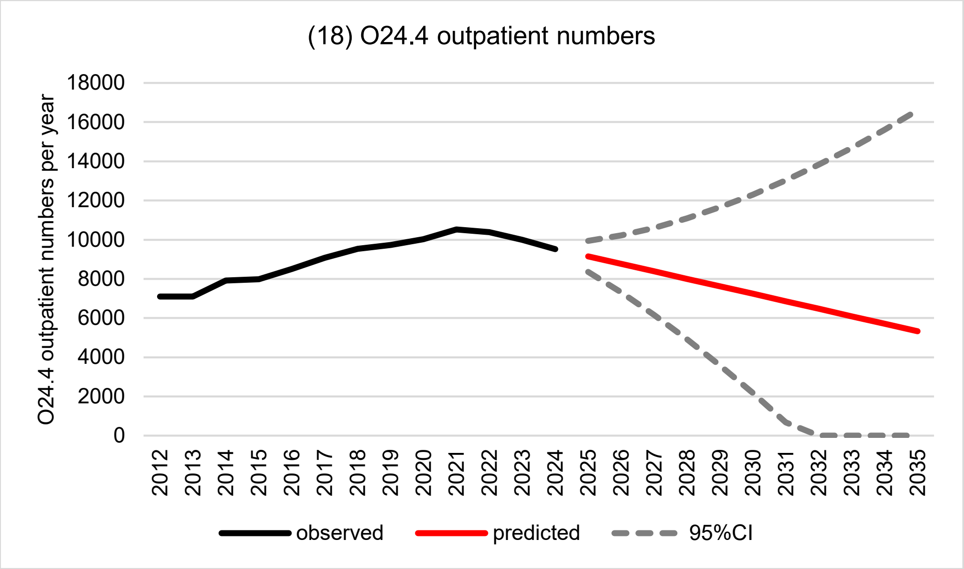

(18) O24.4 outpatient numbers
O24.4 outpatient numbers per year
18000
16000
14000
12000
10000
8000
6000
4000
2000
0
2012
2013
2014
2015
2016
2017
2018
2019
2020
2021
2022
2023
2024
2025
2026
2027
2028
2029
2030
2031
2032
2033
2034
2035
observed
predicted
95%CI

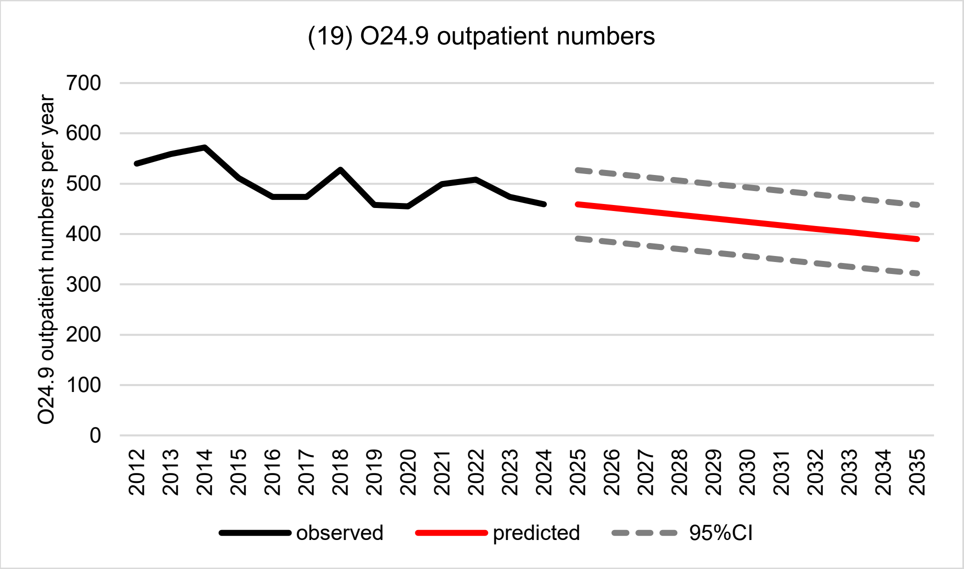

(19) O24.9 outpatient numbers
O24.9 outpatient numbers per year
700
600
500
400
300
200
100
0
2012
2013
2014
2015
2016
2017
2018
2019
2020
2021
2022
2023
2024
2025
2026
2027
2028
2029
2030
2031
2032
2033
2034
2035
observed
predicted
95%CI

Number of FNTMD patients by ICD-10 codes (outpatient care)

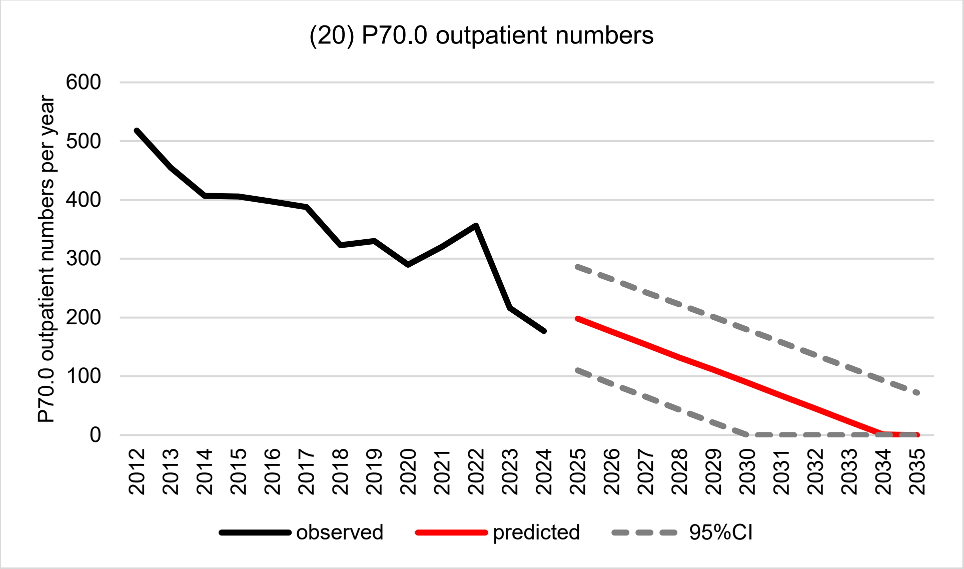

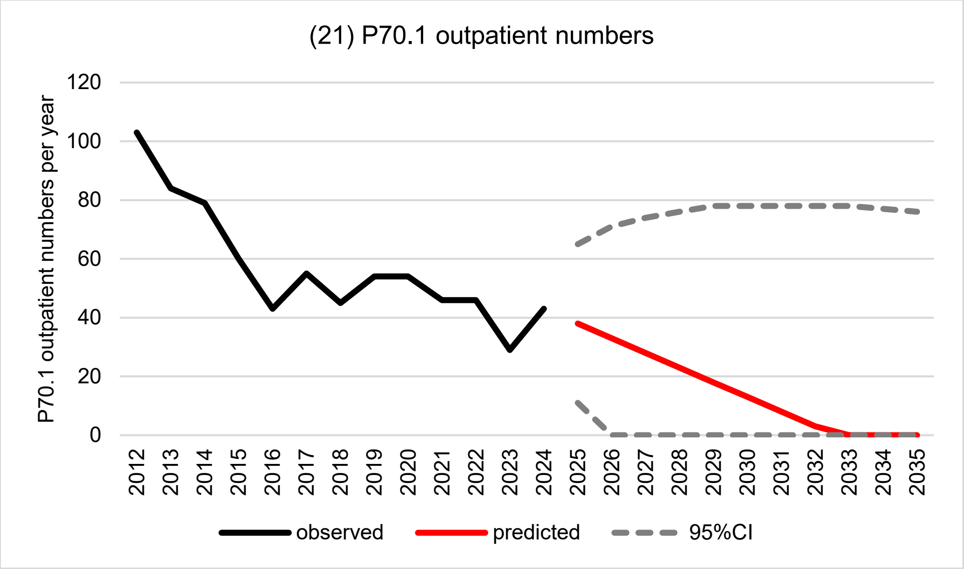

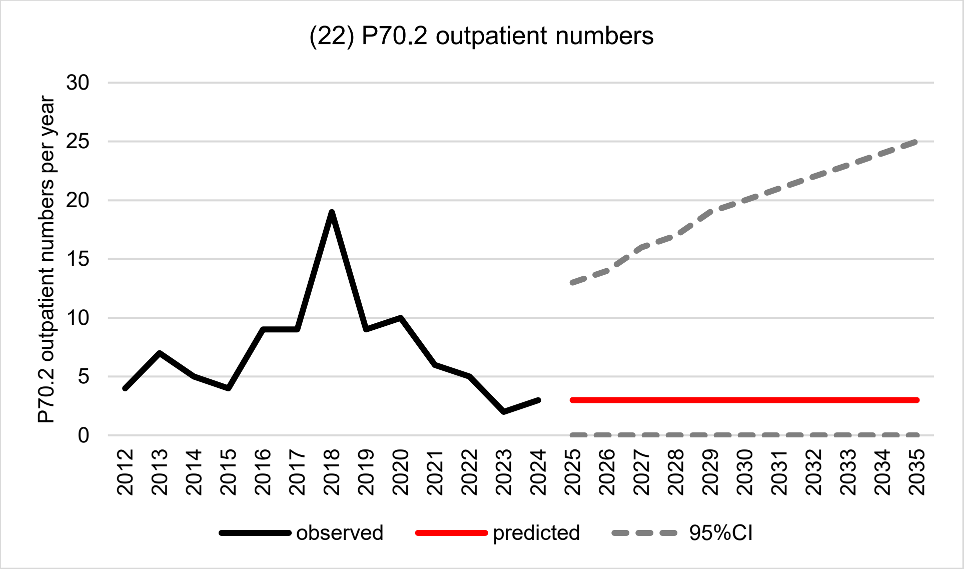

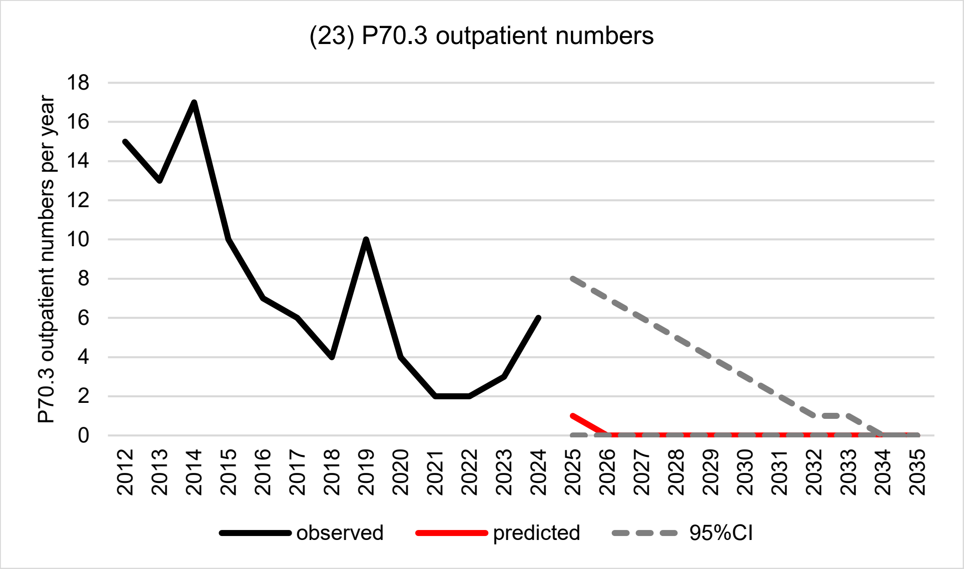
(23) P70.3 outpatient numbers
P70.3 outpatient numbers per year
0
2
4
6
8
10
12
14
16
18
2012
2013
2014
2015
2016
2017
2018
2019
2020
2021
2022
2023
2024
2025
2026
2027
2028
2029
2030
2031
2032
2033
2034
2035
observed
predicted
95%CI

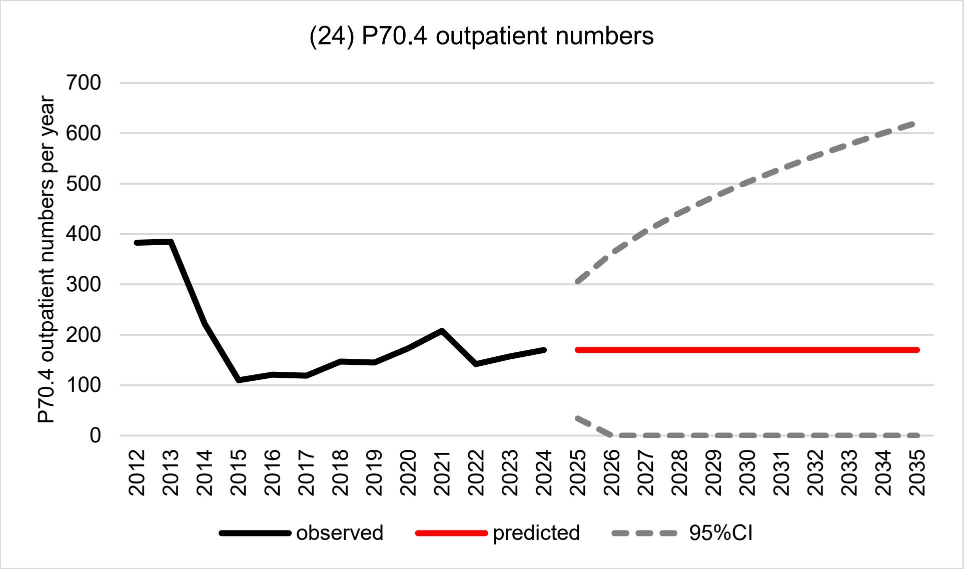
(24) P70.4 outpatient numbers
P70.4 outpatient numbers per year
0
100
200
300
400
500
600
700
2012
2013
2014
2015
2016
2017
2018
2019
2020
2021
2022
2023
2024
2025
2026
2027
2028
2029
2030
2031
2032
2033
2034
2035
observed
predicted
95%CI

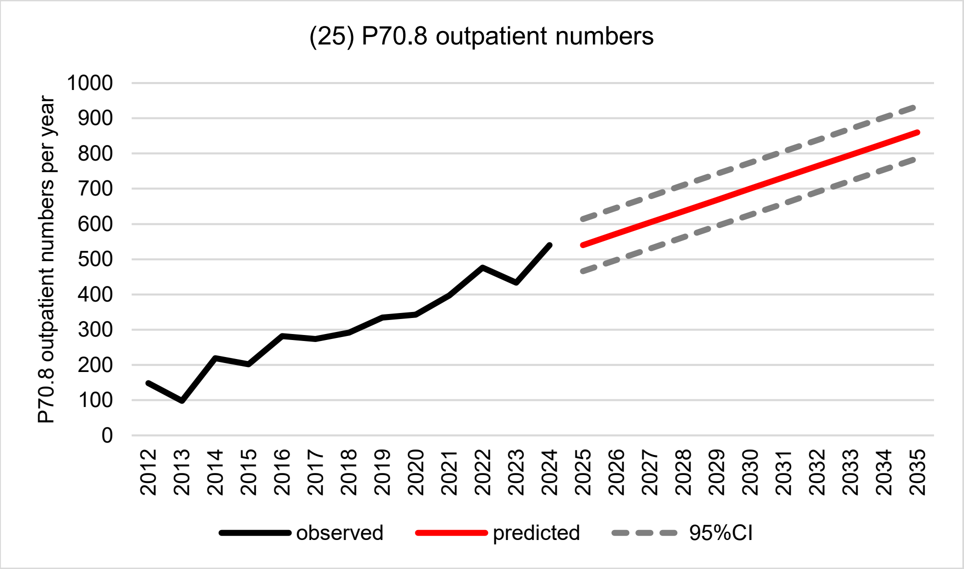
(25) P70.8 outpatient numbers
P70.8 outpatient numbers per year
0
100
200
300
400
500
600
700
800
900
1000
2012
2013
2014
2015
2016
2017
2018
2019
2020
2021
2022
2023
2024
2025
2026
2027
2028
2029
2030
2031
2032
2033
2034
2035
observed
predicted
95%CI

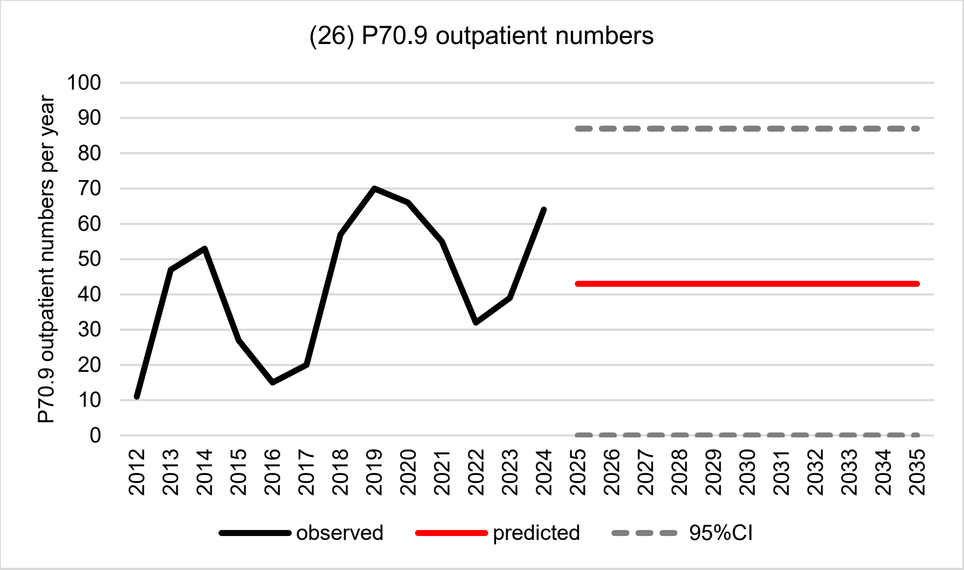

(26) P70.9 outpatient numbers
P70.9 outpatient numbers per year
100
90
80
70
60
50
40
30
20
10
0
2012
2013
2014
2015
2016
2017
2018
2019
2020
2021
2022
2023
2024
2025
2026
2027
2028
2029
2030
2031
2032
2033
2034
2035
observed
predicted
95%CI
