# Supplementary material for: Trends and Projections of the Prevalence of Diabetes Mellitus in Pregnancy and Fetal–Neonatal Metabolic Disorders, 2010–2035: A Nationwide Population-Based Study from Hungary
Source: J Clin Med. 2025 Aug 14;14(16):5740. doi: 10.3390/jcm14165740 (PMC12387990; doi:10.3390/jcm14165740)

Residuals for number of FNTMD patients per thousand live births (inpatient care)

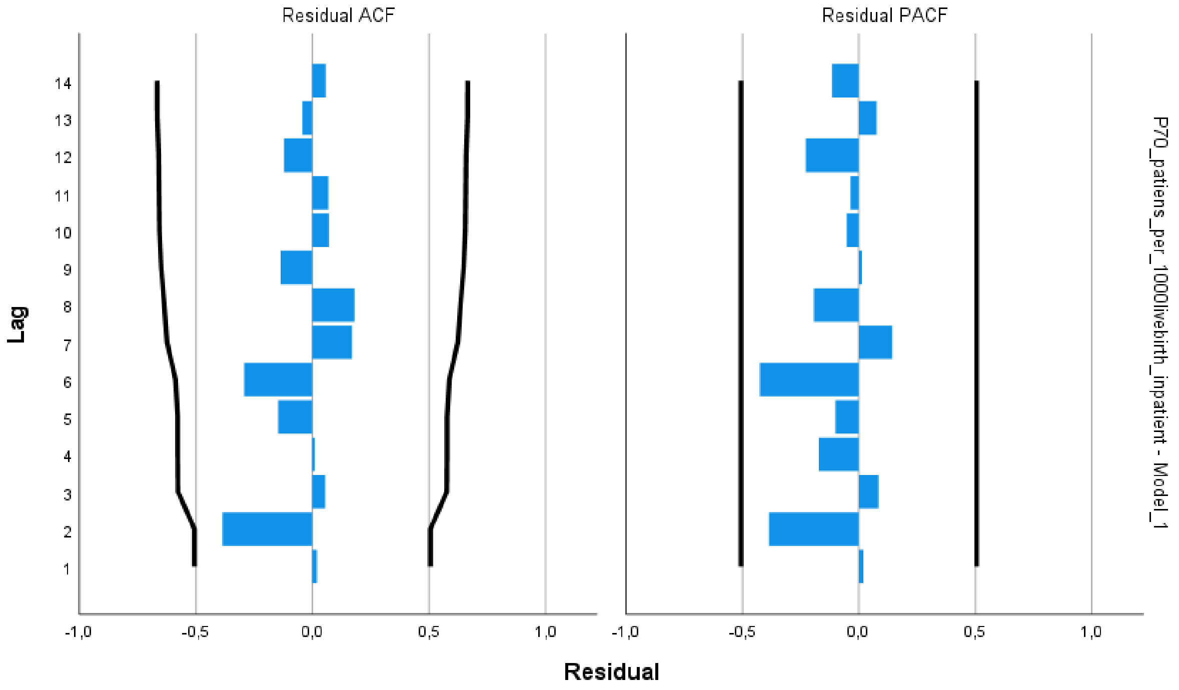

Residuals for number of FNTMD patients per thousand live births (outpatient care)

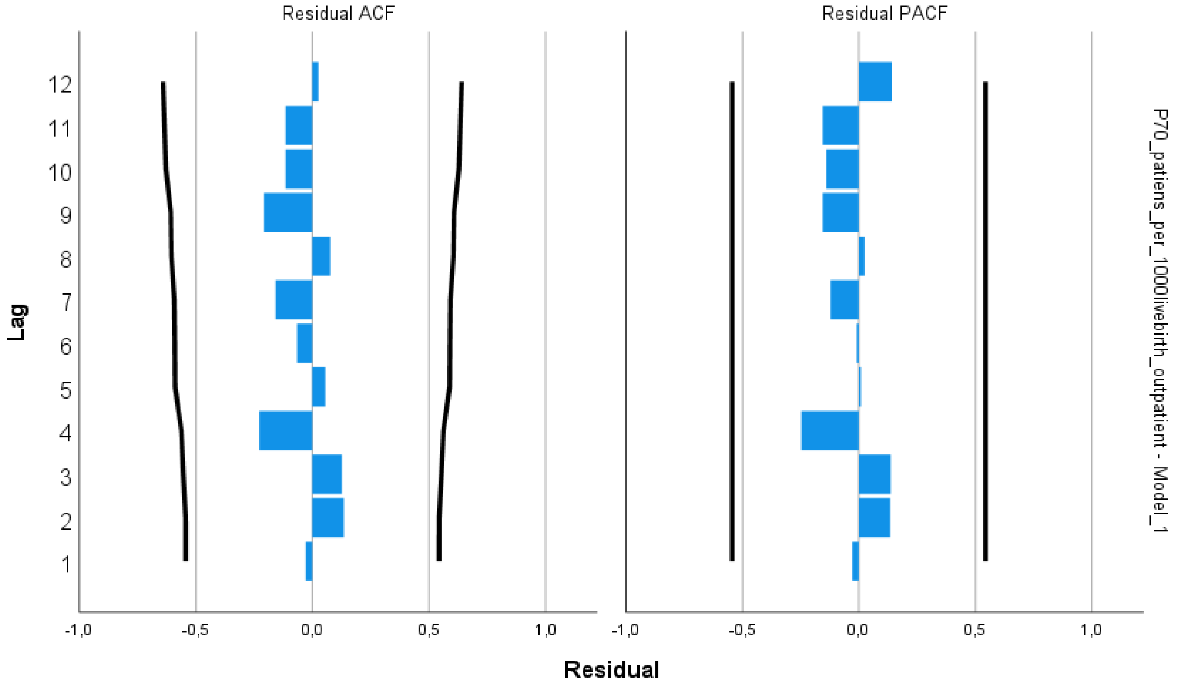

Residuals for number of DMP patients per thousand 15-49-year-old women (inpatient care)

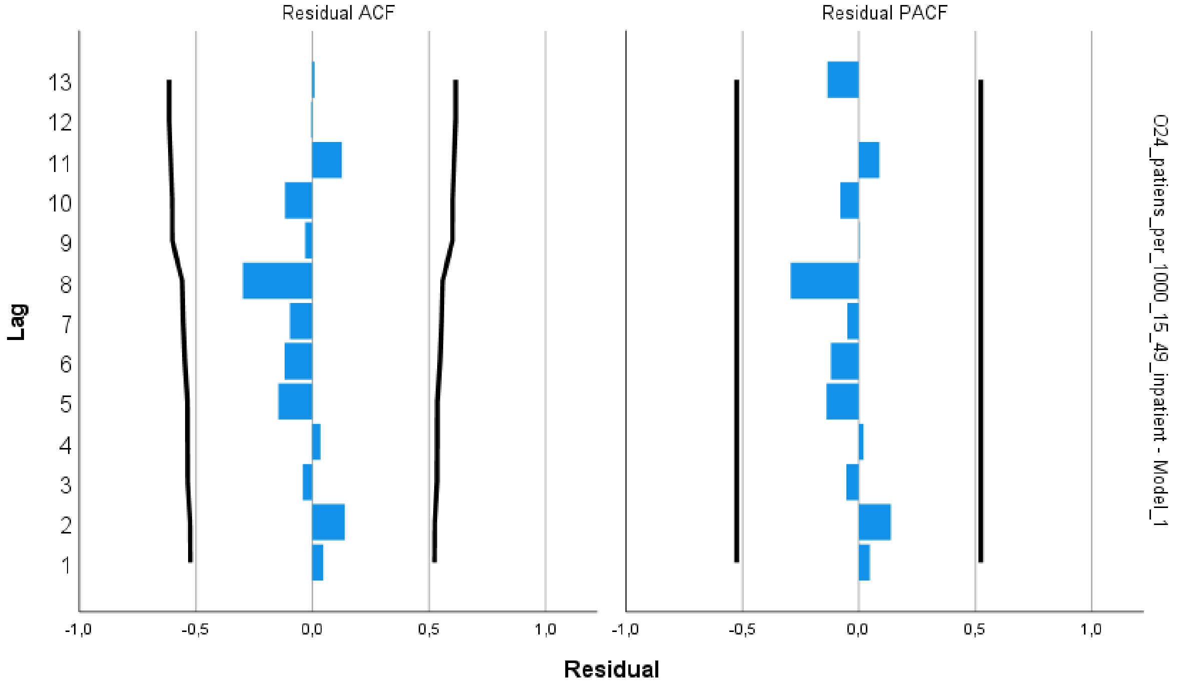

Residuals for number of DMP patients per thousand 15-49-year-old women (outpatient care)

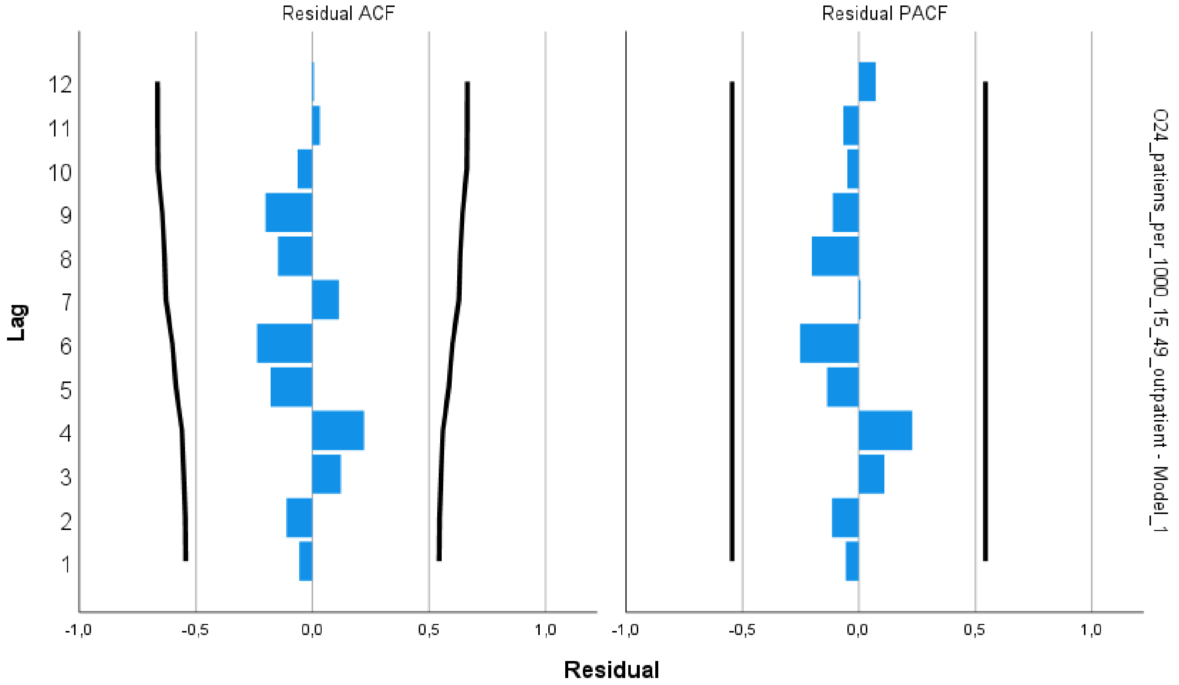

Residuals for crude number of DMP patients (inpatient care)

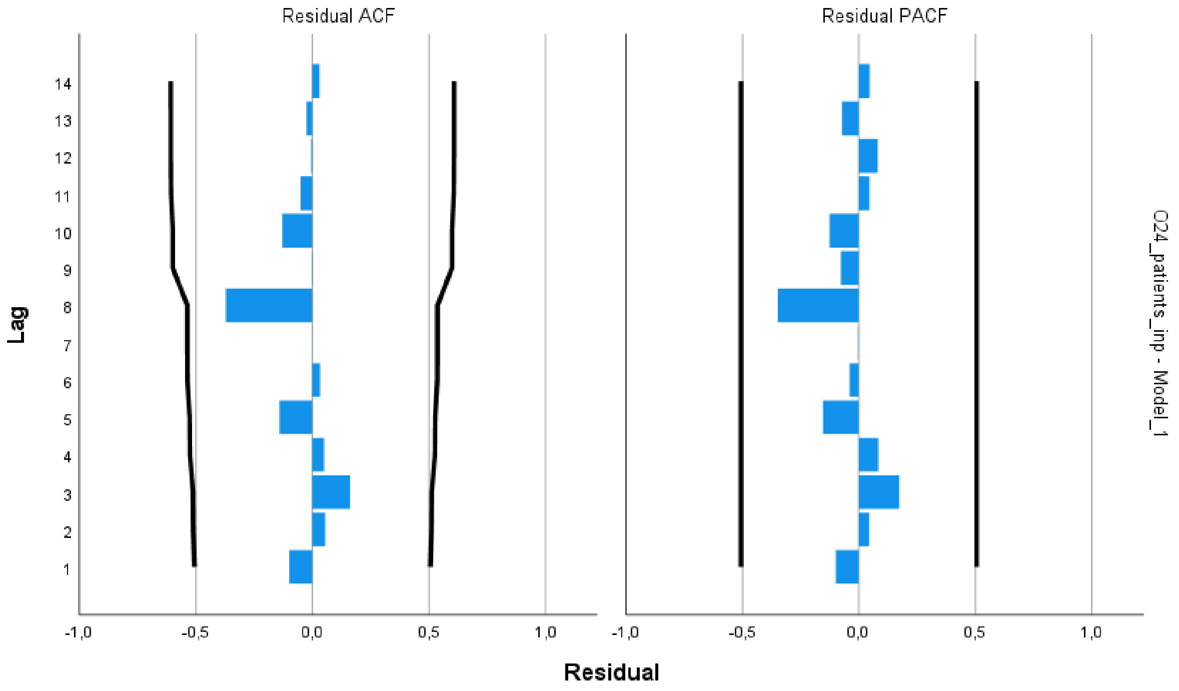

Residuals for crude number of DMP patients (outpatient care)

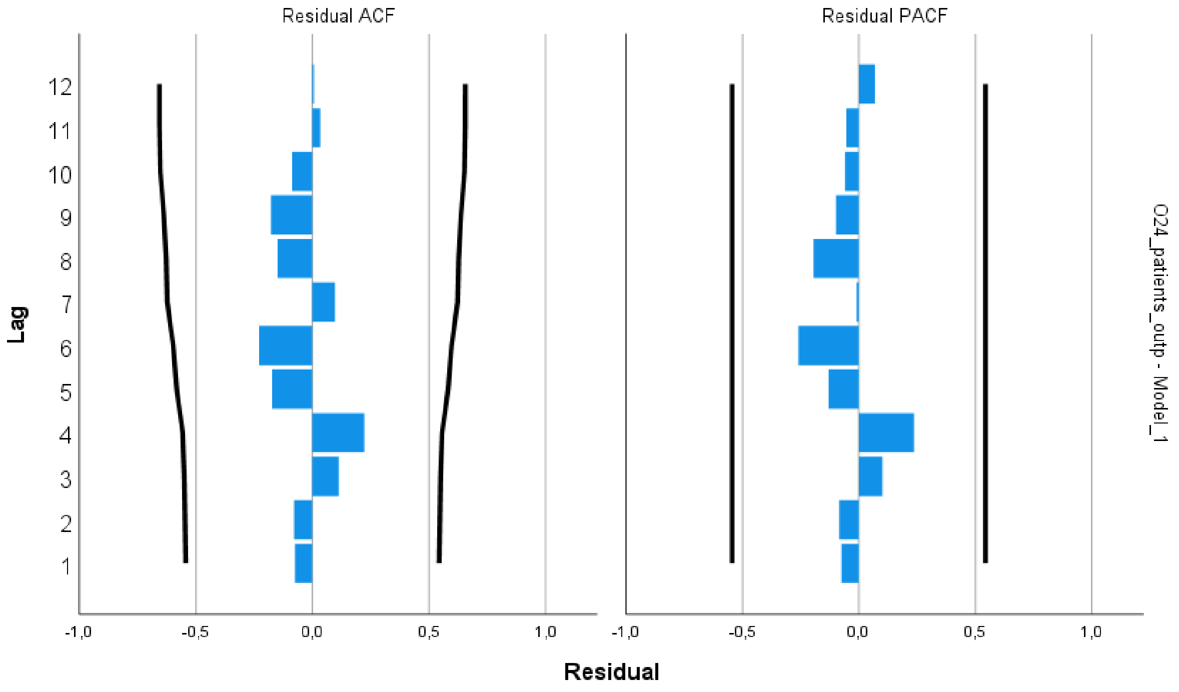

Residuals for crude number of FNTMD patients (inpatient care)

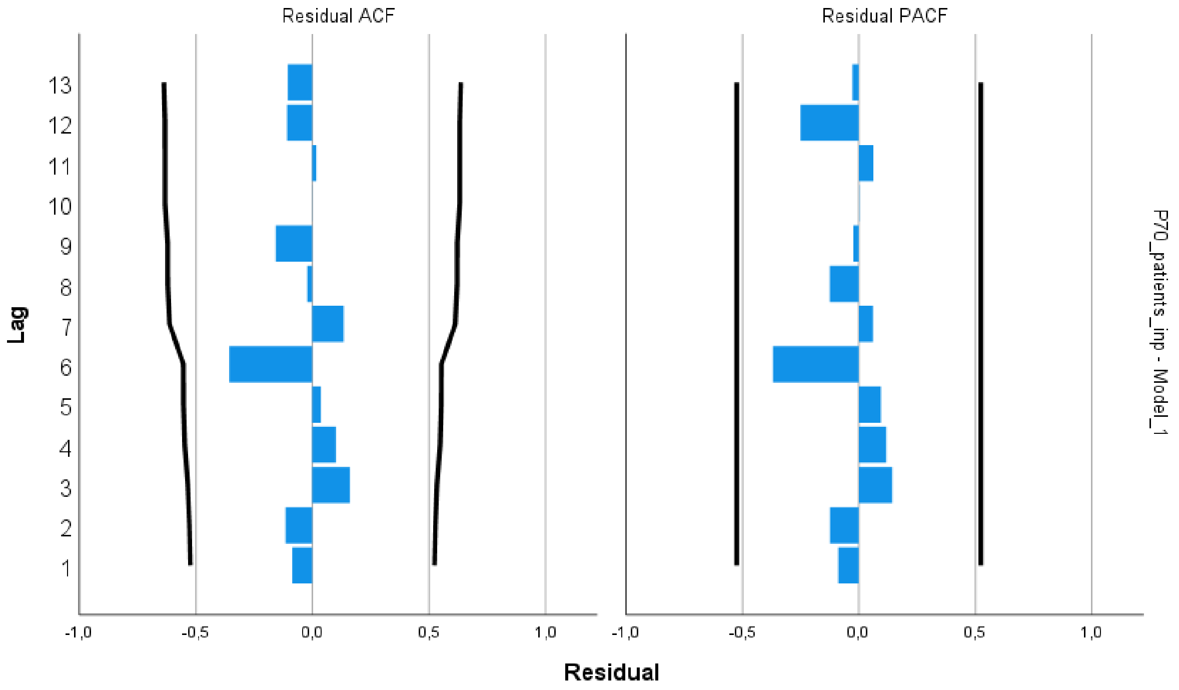

Residuals for crude number of FNTMD patients (outpatient care)

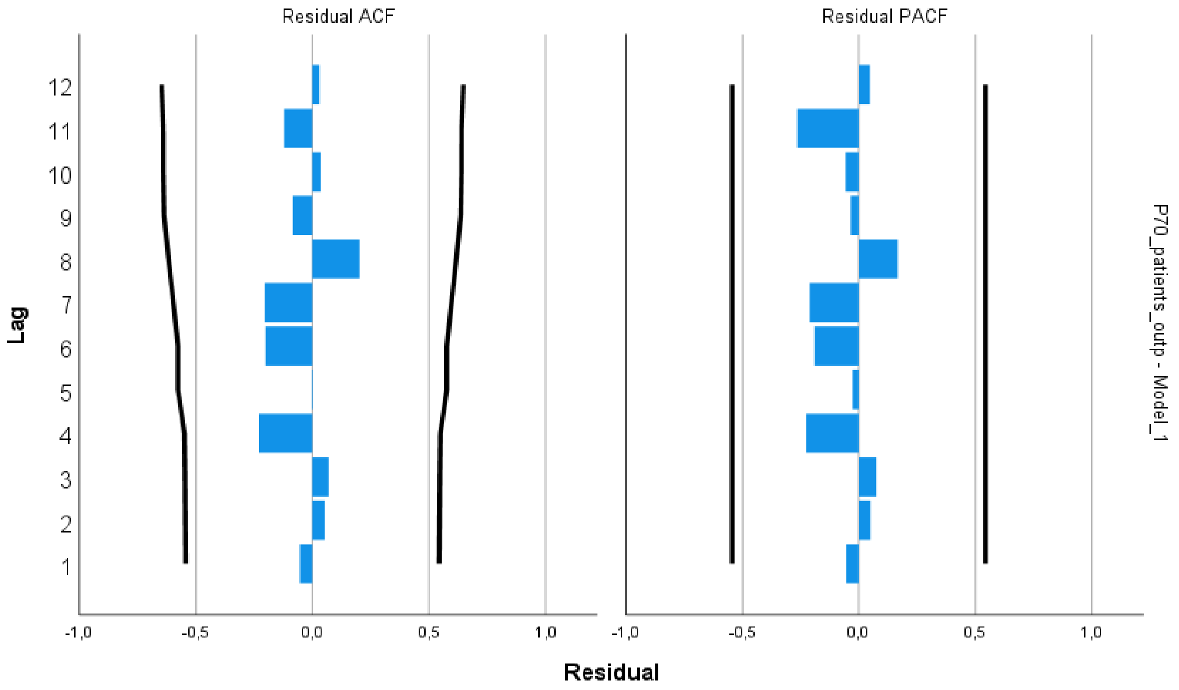

Supplement: Supplementary file 1 [file jcm-14-05740-s001.zip › Csákvári_gest_diabetes_Figure_S1_20250611.pdf]
